# Ultrafast electronic relaxation pathways of the molecular photoswitch quadricyclane

**DOI:** 10.1038/s41557-023-01420-w

**Published:** 2024-02-02

**Authors:** Kurtis D. Borne, Joseph C. Cooper, Michael N. R. Ashfold, Julien Bachmann, Surjendu Bhattacharyya, Rebecca Boll, Matteo Bonanomi, Michael Bosch, Carlo Callegari, Martin Centurion, Marcello Coreno, Basile F. E. Curchod, Miltcho B. Danailov, Alexander Demidovich, Michele Di Fraia, Benjamin Erk, Davide Faccialà, Raimund Feifel, Ruaridh J. G. Forbes, Christopher S. Hansen, David M. P. Holland, Rebecca A. Ingle, Roland Lindh, Lingyu Ma, Henry G. McGhee, Sri Bhavya Muvva, Joao Pedro Figueira Nunes, Asami Odate, Shashank Pathak, Oksana Plekan, Kevin C. Prince, Primoz Rebernik, Arnaud Rouzée, Artem Rudenko, Alberto Simoncig, Richard J. Squibb, Anbu Selvam Venkatachalam, Caterina Vozzi, Peter M. Weber, Adam Kirrander, Daniel Rolles

**Affiliations:** 1 J.R. Macdonald Laboratory, Department of Physics, Kansas State University, Manhattan, KS USA; 2 Physical and Theoretical Chemistry Laboratory, Department of Chemistry, University of Oxford, Oxford, UK; 3 School of Chemistry, Cantocks Close, University of Bristol, Bristol, UK; 4 Chemistry of Thin Film Materials, Friedrich-Alexander-Universität Erlangen-Nürnberg, Erlangen, Germany; 5 European XFEL, Schenefeld, Germany; 6 Istituto di Fotonica e Nanotecnologie (CNR-IFN), CNR, Milano, Italy; 7 Dipartimento di Fisica, Politecnico di Milano, Milano, Italy; 8 Elettra – Sincrotrone Trieste S.C.p.A., Trieste, Italy; 9 Department of Physics and Astronomy, University of Nebraska–Lincoln, Lincoln, NE USA; 10 Istituto di Struttura della Materia (ISM-CNR), CNR, Trieste, Italy; 11 Deutsches Elektronen-Synchrotron DESY, Hamburg, Germany; 12 Department of Physics, University of Gothenburg, Gothenburg, Sweden; 13 Linac Coherent Light Source, SLAC National Accelerator Laboratory, Menlo Park, CA USA; 14 School of Chemistry, University of New South Wales, Sydney, New South Wales Australia; 15 Daresbury Laboratory, Warrington, UK; 16 Department of Chemistry, University College London, London, UK; 17 Department of Chemistry - BMC, Uppsala University, Uppsala, Sweden; 18 Department of Chemistry, Brown University, Providence, RI USA; 19 Max-Born-Institut, Berlin, Germany

**Keywords:** Photochemistry, Chemical physics

## Abstract

The light-induced ultrafast switching between molecular isomers norbornadiene and quadricyclane can reversibly store and release a substantial amount of chemical energy. Prior work observed signatures of ultrafast molecular dynamics in both isomers upon ultraviolet excitation but could not follow the electronic relaxation all the way back to the ground state experimentally. Here we study the electronic relaxation of quadricyclane after exciting in the ultraviolet (201 nanometres) using time-resolved gas-phase extreme ultraviolet photoelectron spectroscopy combined with non-adiabatic molecular dynamics simulations. We identify two competing pathways by which electronically excited quadricyclane molecules relax to the electronic ground state. The fast pathway (<100 femtoseconds) is distinguished by effective coupling to valence electronic states, while the slow pathway involves initial motions across Rydberg states and takes several hundred femtoseconds. Both pathways facilitate interconversion between the two isomers, albeit on different timescales, and we predict that the branching ratio of norbornadiene/quadricyclane products immediately after returning to the electronic ground state is approximately 3:2.

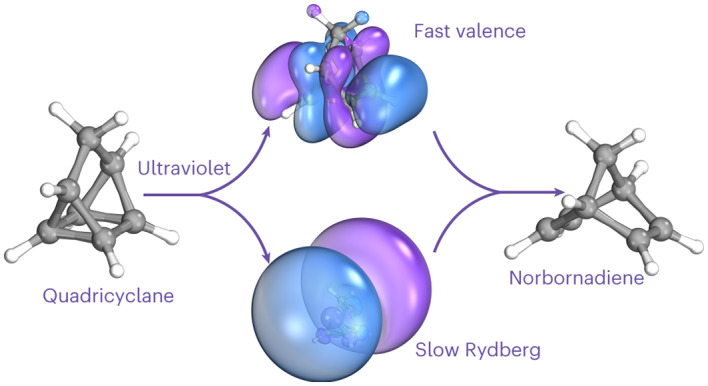

## Abstract (web summary)

Light-induced ultrafast switching between the molecular isomers norbornadiene and quadricyclane can reversibly store and release a substantial amount of chemical energy. Two competing pathways have now been identified by which electronically excited quadricyclane molecules relax to the electronic ground state, facilitating interconversion between the two isomers on different timescales.

## Main

**Fig. 1:**
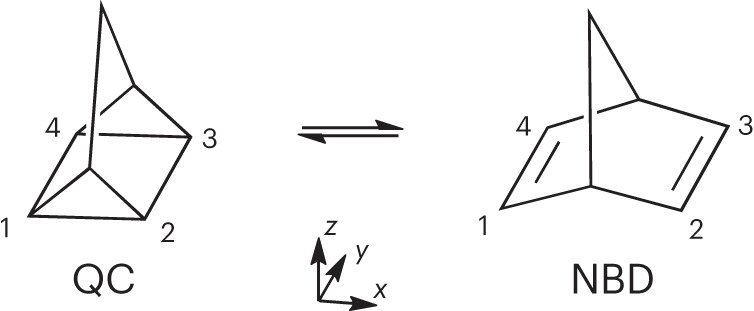
Schematic of the QC ⇄ NBD interconversion. The isomerization reaction breaks (or forms) the 1–2 and 3–4 carbon–carbon bonds in QC, with the energy of the QC ground state located ~1 eV above the ground state of NBD. The interconversion can be driven by light absorption, allowing the system to act as a molecular photoswitch. Source data

Molecular photoswitches are an exciting area of research with emerging applications in catalysis, photochromic materials, molecular machines, logic devices and energy storage^1^. The last example includes molecular solar thermal (MOST) energy storage solutions, whereby molecular isomers are exploited to absorb, store and later release solar energy as heat^2,3^. An important model system for such applications is the photoswitchable pair of isomers quadricyclane (QC), a highly strained multicyclic hydrocarbon, and its lower-energy isomer norbornadiene (NBD)^4–9^, whose International Union of Pure and Applied Chemistry (IUPAC) names are tetracyclo[3.2.0.0^2,7^.0^4,6^]heptane (QC) and bicyclo[2.2.1]hepta-2,5-diene (NBD). The isomers, shown in Fig. 1, interconvert upon photoabsorption in the ultraviolet (UV) range^10–13^. More broadly, the photoisomerization of NBD → QC is an example of a [2 + 2] cycloaddition reaction, and the behaviour of the molecule is reminiscent of the prototypical ethylene dimerization, with the bridging framework acting to hold the ethylene moieties together^14,15^. We investigate the mechanism of the reverse interconversion, QC → NBD, which is of both fundamental photochemical interest and practical importance since it represents the undesired UV-induced photoreversion process in QC/NBD-derived MOST systems.

Early time-resolved ion mass spectrometry experiments on gas-phase NBD noted signatures of ultrafast molecular dynamics upon UV excitation at 200 nm (6.20 eV), which were speculatively attributed to transitions from a superposition of Rydberg and valence states to the electronic ground state via a series of conical intersections on a sub-100 femtosecond timescale^15^. A subsequent study on QC excited at 208 nm (5.96 eV) employed time-resolved Rydberg fingerprint photoelectron spectroscopy^16^. The latter work suggested that QC isomerizes to NBD on the 3*s* Rydberg state within 140 fs and that relaxation to the electronic ground state occurs within 400 fs. Notably, that study could not observe the dynamics all the way to the ground state since the probe photons had insufficient energy to ionize ground-state molecules. Theoretical work has focused on the photorelaxation of NBD, examining the electronic structure^4,9,17^ and the dynamics^18,19^. In both cases, the key role of interactions between the Rydberg manifold and valence states in NBD was highlighted.

In the present study, we use time-resolved photoelectron spectroscopy (TRPES) with extreme ultraviolet (XUV) probe pulses, capable of tracking the entire reaction path from the initial UV-excited states through to the electronic ground states of both isomers. TRPES with XUV pulses is a powerful method for mapping complex chemical dynamics^20–24^. Our experimental data, supported by high-level simulations, provide a much more complete description of the deactivation mechanisms in QC, thus providing mechanistic insights that can be used to design more-efficient MOST systems.

## Results

**Fig. 2:**
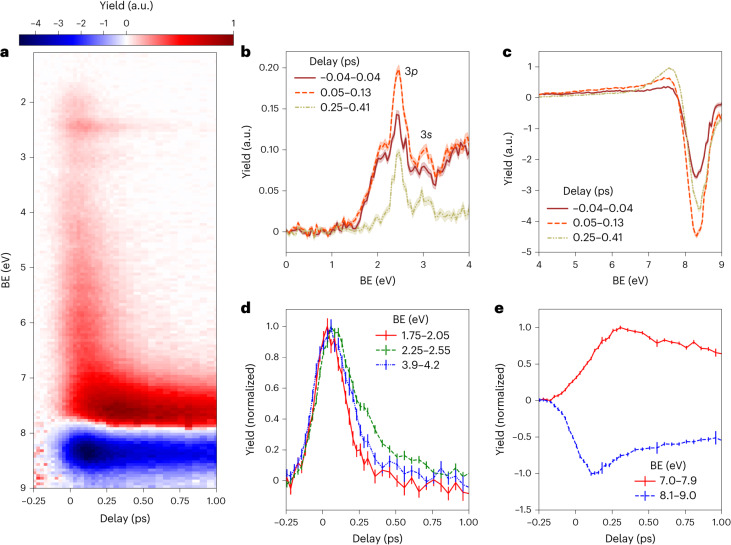
Experimentally measured TRPES spectra of UV-excited QC. **a**, Photoelectron spectra as a function of BE (with respect to the first ionization potential, D_0_) and pump–probe time delay between UV and XUV pulses, plotted as the electron yield difference between spectra taken with and without the UV-excitation pulse (Extended Data Fig. 1). The respective electron yield at each delay is normalized to the FEL pulse energy before subtraction, and the difference signal is normalized to the integrated electron yield without UV excitation, both summed over all shots at the respective delay. Negative delays correspond to the FEL pulse preceding the UV excitation pulse. **b**,**c**, Photoelectron BE spectra in the excited-state region (**b**) and at large BE values that sample the electronic ground state (**c**) at specific time delays. **d**,**e**, Delay-dependent electron yield in the denoted BE regions, normalized to have the same peak amplitudes. The data and error bands (shading) in **b** and **c**, and the error bars in **d** and **e**, represent the mean value of approximately 40,000 single-shot digitizer traces and the 68% confidence interval obtained from a bootstrapping analysis (Methods). Source data

We initiate the photochemical reaction in gas-phase QC by a UV excitation pump pulse at 200.6 nm (6.18 eV), followed by an XUV ionization probe pulse at 18.97 eV (65.35 nm) from a seeded free-electron laser (FEL). The TRPES spectra obtained as the difference between the spectra acquired with and without the pump pulse (Methods and Supplementary Section 1.4 for details) are shown as a two-dimensional false-colour map in Fig. 2a. One-dimensional spectra obtained by averaging over selected time-delay ranges are displayed in Fig. 2b,c (additional spectra are shown in Extended Data Fig. 2). Several prominent features are apparent. A narrow and long-lived feature at ~2.3 eV binding energy (BE), visible as a faint horizontal line in Fig. 2a and as a clear peak in Fig. 2b, is assigned to two of the three 3*p* Rydberg states of QC in accordance with the literature^16^. The 3*s* Rydberg state can be seen at a BE of ~2.9 eV in Fig. 2b but has a weaker signature than in earlier studies^16^, suggesting less excitation to the 3*s* state at 200.6 nm compared to 208 nm. Figure 2a also shows a spectrally broad and short-lived vertical feature in the BE range of ~1.5–7 eV, which merges into a long-lived horizontal band at ~7–8 eV BE, both of which were not observed in the earlier work that did not have a sufficient photon energy to ionize this BE region. These observations suggest the existence of a rapid (<100 fs) deexcitation pathway leading to the formation of vibrationally hot photoproducts, while the longer timescale of the Rydberg-associated features indicates the existence of a second, slower decay mechanism, which corresponds to the pathway identified in earlier work^16^. Finally, there is a pronounced negative signal in the difference spectrum in the BE range 8–9 eV, assigned to the depletion of the QC ground state, followed by a partial recovery.

Delay-dependent photoelectron yields in selected spectral regions are shown in Fig. 2d,e. They display a nearly Gaussian shape at low BE values with a temporal width that is mostly given by the instrument response function (Methods), while clear exponential ‘tails’ are visible in the range of the Rydberg excitations and in the high-BE yields. Least-square fits of the delay-dependent yields are provided in Supplementary Figs. 9–11.

**Fig. 3:**
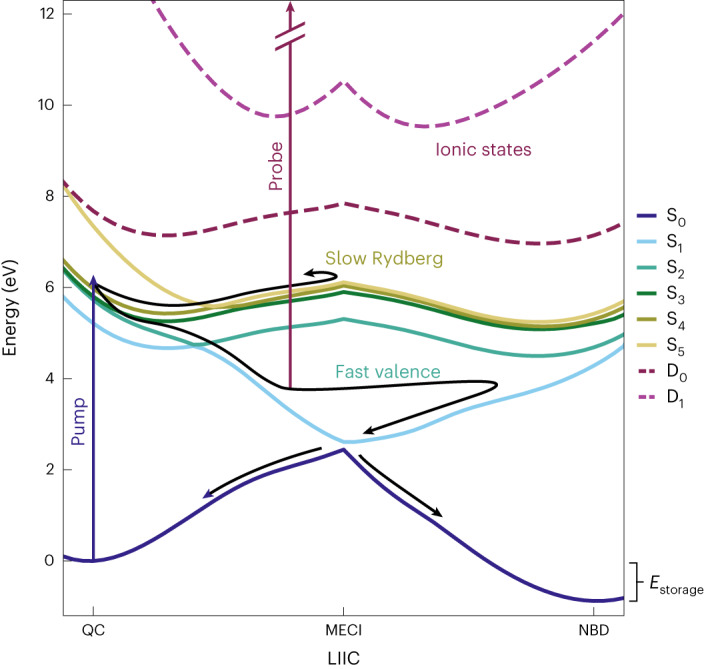
Potential energy curves for QC and NBD along the LIICs. Potential energy curves for the ground state S_0_ and excited states S_1_–S_5_, as well as the cation ground state D_0_ and first excited state D_1_ (dashed lines), calculated along the LIICs, first from QC to the S_1_/S_0_ MECI and then from the MECI to NBD, using RMS-CASPT2(2,6)/6-31G* + D (Methods and Supplementary Section 2.1). The two proposed reaction pathways are indicated schematically by black arrows, with the slow Rydberg pathway supporting vibrational motions on the Rydberg states before relaxing via the S_1_ state, while the fast valence pathway descends on S_1_, bypassing the MECI on the initial descent, before crossing onto the S_0_ ground state to form both products. The molecular geometry at the MECI has a distinct rhombic distortion (Extended Data Fig. 3). The *E*_storage_ indicates the energy stored in QC relative to NBD (~1 eV). Corresponding vertical excitation energies are listed in Table 1, and a plot including higher excited states and the approximate state characters is provided in Extended Data Figure 4. Source data

The nature of the previously unreported broad, short-lived feature with BE values in the range ~1.5–7 eV is investigated further using high-level electronic structure calculations and non-adiabatic dynamics simulations. Figure 3 shows calculated potential energy curves for the ground (S_0_) and first five singlet excited (S_1_–S_5_) electronic states identified as being active in the dynamics probed by the present experiment, plotted along a linear interpolation in internal coordinates (LIIC) from the equilibrium geometry of QC(S_0_) to the S_1_/S_0_ minimum energy conical intersection (MECI) and then continued along a second LIIC from this MECI to the NBD(S_0_) equilibrium geometry. The dominant electronic characters and adiabatic energies at the equilibrium geometries and the MECI are given in Table 1. In the Franck–Condon (FC) region of QC, the S_1_ adiabatic state has 3*s* Rydberg character; S_3_ and S_4_ are 3*p* Rydberg states; and S_2_ and S_5_ are a pair of mixed 3*p*_*x*_/valence (V) states. As the QC molecule approaches the MECI geometry, the S_1_ state acquires valence character, which persists into the FC region of NBD. A complete discussion of the vertical electronic spectra of both isomers, complementing the work by Palmer et al.^17,25^, is reserved for a separate publication^26^.

**Table 1:** Vertical excitation energies for each of the five excited singlet states, labelled S_1_–S_5_, with the dominant electronic character provided in parentheses, as well as the S_0_ → D_0_ ionization potential at the respective equilibrium geometries of QC and NBD and at the S_1_/S_0_ MECI geometry, calculated using RMS-CASPT2(2,6)/6-31G* + D

State	QC (eV) (character)	MECI (eV) (character)	NBD (eV) (character)
S_1_	5.29 (3*s*)	0.00 (V)	5.25 (V)
S_2_	5.82 (3*p*_*x*_/V)	2.74 (3*s*)	5.61 (3*s*)
S_3_	5.88 (3*p*_*y*_)	3.31 (3*p*_*x*_)	6.11 (3*p*_*y*_)
S_4_	6.05 (3*p*_*z*_)	3.47 (3*p*_*y*_)	6.28 (3*p*_*x*_)
S_5_	7.53 (3*p*_*x*_/V)	3.53 (3*p*_*z*_)	6.34 (3*p*_*z*_)
IP (S_0_ → D_0_)	7.67	5.26	8.02

Note that the numbering of the states is directly related to the electronic structure model used. The geometries are shown in Extended Data Fig. 3. The value of the average carbon–carbon distance coordinate *r*_CC_ for the three geometries is ${r}_{{{\mathrm{CC}}}}^{{{\mathrm{QC}}}}$ = 1.51 Å, ${r}_{{{\mathrm{CC}}}}^{{{\mathrm{MECI}}}}$ = 1.98 Å and ${r}_{{{\mathrm{CC}}}}^{{{\mathrm{NBD}}}}$ = 2.47 Å. At the QC ground-state geometry, in the two mixed states S_2_ and S_5_, the 3*p*_*x*_ component is dominant in S_2_ and the valence (V) component is dominant in S_5_.

## Discussion

The potential energy curves in Fig. 3 suggest the possible mechanisms that underpin the features observed in the TRPES data, with the two pathways indicated schematically by black arrows. From the QC ground state, the bandwidth of the pump pulse (0.6 nm) allows excitation to a manifold of closely spaced states with 3*p* Rydberg character (Extended Data Fig. 7). Population in the 3*p*_*y*/*z*_ Rydberg states (S_3_ and S_4_ in the FC region) then evolves via a (comparatively) slow Rydberg pathway on the rather flat Rydberg manifold at excitation energies of ~6 eV; we anticipate that this would correspond to the pathway observed previously^16^. Due to the similar topographies of the Rydberg and ground-state ion (D_0_) adiabatic potential energy surfaces, the BE values for the slow Rydberg pathway can be expected to be relatively constant—as observed experimentally. Conversely, in the fast valence pathway, the population in the strongly mixed Rydberg–valence 3*p*_*x*_/V state (S_2_ in the FC region) is subject to efficient non-adiabatic coupling to S_1_, funnelling down towards a conical intersection with the S_0_ state. In the proposed model, the BE values for the fast valence pathway would increase rapidly, consistent with the short-lived structure observed for experimental BE values in the range of ~1.5–7 eV.

**Fig. 4:**
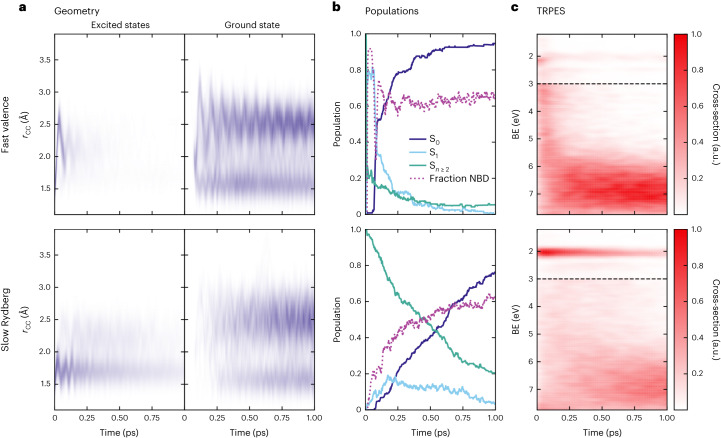
Simulated dynamics of UV-excited QC. The simulations are separated into the fast valence mechanism (top row, corresponding to trajectories starting on the S_2_ state) and the slow Rydberg mechanism (bottom row, trajectories originating on the S_3/4_ Rydberg states). A zoomed-in depiction of the first 200 fs is shown in Extended Data Fig. 6. **a**, False-colour maps for average carbon–carbon distance *r*_CC_ = (*r*_12_ + *r*_34_)/2 versus time (Methods). The left and right sides show the nuclear dynamics in the excited states and the ground state (S_0_), respectively. **b**, Populations of the electronic states as a function of time, where S_*n* ≥ 2_ shows the cumulative population of the excited states other than S_1_. These plots also show the fraction of molecules with NBD-like geometry (defined as *r*_CC_ > 2.0 Å). **c**, Simulated TRPES data, convoluted by Gaussian functions with full-width at half-maximum of 0.1 eV and 94 fs. Signals with BE > 3 eV (below the horizontal dashed line) have been multiplied by a factor of three prior to display. Source data

We confirm these hypothesized mechanisms using non-adiabatic mixed quantum–classical trajectory simulations (Methods). The simulations divulge that the photodynamics of excited QC are strongly dependent on the initial excited state. Figure 4 shows the results separated into two sets. Dynamics initiated on the S_2_ (3*p*_*x*_/V) state are labelled fast valence, shown in the top row, while dynamics initiated on the S_3_ and S_4_ (3*p*_*y*_ and 3*p*_*z*_) states are labelled slow Rydberg and shown in the bottom row. The plots in Fig. 4a show false-colour maps of the nuclear dynamics along the coordinate associated with the making and breaking of the 1–2 and 3–4 carbon–carbon bonds (r_12_ and r_34_, respectively) during the interconversion between QC and NBD (Methods). The left and right sides show the trajectories on the excited states and ground state, respectively. Figure 4b shows the time-dependent electronic-state populations and the fraction of population best defined as NBD-like, while the false-colour maps in Fig. 4c finally show the contributions to the simulated TRPES spectrum to aid comparison with the experimental data in Fig. 2a.

We first examine the simulation results for the fast valence mechanism shown in the top row of Fig. 4, corresponding to trajectories initiated on the S_2_ (3*p*_*x*_/V) state. One-photon excitation to the 3*p*_*x*_/V state from the S_0_ state is symmetry forbidden at the exact equilibrium geometry, but the transition gains strength from the Herzberg–Teller effect (45% excitation fraction; Methods). The nuclear dynamics in the electronic excited states (Fig. 4a, left side) break the carbon–carbon bonds along *r*_12_ and *r*_34_, allowing the molecule to deform towards NBD-like geometries (*r*_CC_ =(r_12_ + r_34_)/2 > 2.0 Å). The wave packet then returns towards QC-like geometries (smaller *r*_CC_) and crosses to the S_0_ ground state via a conical intersection, appearing on the right side. It immediately partitions into QC-like and NBD-like geometries. The recurrences in the upper map reflect the persistence of the ‘butterfly’-like vibrational motion of the NBD products when they first reach the S_0_ state (with wave number $\widetilde{\upsilon }$ ≈ 428 cm^–1^)^27^. The corresponding motion in QC has a higher wave number ($\widetilde{\upsilon }$ ≈ 721 cm^–1^)^28^ but can also be discerned in the lower maps.

This picture is confirmed in the upper plot of Fig. 4b, which shows almost immediate population transfer from S_2_ to S_1_ via internal conversion (zoomed-in view in Extended Data Fig. 6), followed by another rapid internal conversion to S_0_, with only a small fraction (<10%) of the population remaining in the Rydberg manifold after 1 ps. The time-dependent fraction of molecules with NBD-like geometries (that is, with *r*_CC_ > 2.0 Å) in the same graph shows a rapid early increase and then decline (mirroring the S_1_ state population), finally converging to a branching ratio of ~3:2 for NBD-like to QC-like geometries in the S_0_ state. This ratio is set by the decay dynamics rather than energetics, as the timescales are far too short for thermalization on the ground state.

The theoretically predicted TRPES signal in Fig. 4c shows a curved band of intensity evolving from the BE values of the Rydberg state (~2.1 eV) down to that of the S_0_ state (~7 eV), in good accord with the ‘hockey-stick’-shaped feature in the experimental data (Fig. 2a). In part, this broad feature reflects the very fast motion of the wave packet down the 3*p*_*x*_/V surface, with a corresponding rapid increase in the BE with respect to D_0_. In addition, there is a smaller contribution from the D_1_ ionization channel when the wave packet approaches the MECI. Figure 4a (upper left map) shows that the bond breaks and reforms in under 100 fs, faster than the instrument response function of the experiment, making these features appear almost simultaneously at time *t* = 0. A more detailed analysis of the components of the photoelectron signal is provided in the Supplementary Information.

Next, we consider the slow Rydberg pathway with trajectories initiated on the S_3/4_ (3*p*_*y*/*z*_) states, shown in the bottom row of Fig. 4. Already on the left side of Fig. 4a, it is apparent that the excited-state dynamics persist for substantially longer than in the fast valence mechanism, and that the dynamics are more centred at QC-like molecular geometries (*r*_CC_ < 2.0 Å), with only a small fraction of the trajectories exploring NBD-like geometries in the 3*p* excited states, discernable as a faint signal at *r*_CC_ > 2.0 Å. Examining the populations in Fig. 4b, we see a comparatively slow but steady deactivation of the Rydberg manifold states (S_2_ and higher) with a matching increase in S_0_ population. The decay proceeds via the same 3*p*_*x*_/V state as the fast valence mechanism, evidenced by the build-up of population in the S_1_ intermediate state, which acts as a funnel towards the S_0_ state via the conical intersection. It appears that this conical intersection has a strong influence on the NBD/QC branching ratio in the S_0_ state, since the fraction of NBD (Fig. 4b, bottom) approaches the same value as the fast valence pathway.

The simulated TRPES signal in Fig. 4c shows a long-lived feature with BE ≈ 2.1 eV, corresponding to the 3*p* Rydberg states (with potentials along the LIIC of interest that closely track that of the D_0_ ionic state), in good accord with the experimental data, which show the corresponding feature attributable to ionization from these Rydberg states at BE ≈ 2.3 eV (Fig. 2a). At long times, the TRPES signal seen in the slow Rydberg and fast valence dynamics are essentially identical. Both pathways involve similar ultimate decays to the S_0_ state, yielding similar branching ratios of hot QC and NBD products. Note that the vertical ionization potentials of QC and NBD are similar, differing by ~0.3 eV (Methods). Thus, the TRPES signals of hot ground-state QC and NBD species overlap, and the experimentally observed bleach recovery in Fig. 2e includes components from both isomers.

Notwithstanding the very good agreement between experiment and theory, some discrepancies are evident. The simulations return longer Rydberg state lifetimes than observed experimentally, which could reflect shortcomings in the electronic structure theory, for example, regarding the strength and location of the couplings with the 3*p*_*x*_/V state, additional decay paths not accounted for by the active space used or the accuracy of the predicted ionization cross-sections.

In summary, TRPES studies using XUV photons from a seeded FEL have provided new and detailed insights into the relaxation pathways of electronically excited QC molecules formed by photoexcitation at 200.6 nm, from Rydberg states through to vibrationally excited hot ground-state QC and NBD molecules. We present observations of the dominant relaxation pathway in QC, involving strong Rydberg–valence coupling, alongside the previously observed Rydberg-state-mediated isomerization^16^. The present combined experimental and theoretical study shows that all three 3*p* Rydberg states of QC are excited by the 200.6 nm pump pulse. The lowest-energy (3*p*_*x*_) state is strongly coupled to a valence state, giving rise to the fast valence pathway characterized by ultrafast electronic decay and nuclear motion. The other two support initial nuclear dynamics in the Rydberg manifold, prior to comparatively slower decay to the ground state (identified here as the slow Rydberg pathway). The simulations suggest that ~40% of the vibrationally excited ground-state products sampled at pump–probe delays of ~1 ps correspond to QC-like structures, irrespective of the decay pathway followed, and that this early-time partitioning is determined by the molecular dynamics rather than thermodynamics.

The insights provided by the present study point to key features that affect the photodynamics: the partial absorption cross-sections, the strength of the non-adiabatic couplings to the valence excited state and the relative stabilities and geometries of the regions of maximal interstate coupling, that is, the conical intersection between the S_1_ and S_0_ states. For potential applications, these properties could be manipulated using substituent groups, using spatial confinement and/or by the choice of excitation wavelength^5,8,19,29^. For instance, the detailed nature of valence/Rydberg mixing in the valence state controls its energy and accessibility, which could be used to affect the system’s function as a molecular photoswitch. The manner in which the wave packet approaches the S_1_/S_0_ conical intersection region is also important, and this could be manipulated either in the preparation of the wave packet or by altering the position and shape of the conical intersection.

From a broader photochemical perspective, one might conjecture that the bipartite dynamics found here will be a common feature whenever a manifold of Rydberg states intersects valence states in the FC region^30–32^. The excitation energies to Rydberg states typically fall within ~3 eV of the associated ionization limit, *E*^IP^ (IP, ionization potential), and, in most small to medium-sized molecules, will overlap with those of one or more excited valence states. Rydberg excitations will manifest as a longer-lived trapping component, whereas excitations to valence states (or, as here, to a mixed Rydberg–valence state) will lead to more-rapid relaxation that gives rise to broad features in the photoelectron spectrum. Broadband excitation can excite both types of states, leading to both types of mechanism. Bifurcation of wave packets has been reported previously, for example, in the photoisomerization of stilbene^33^ and in photoexcited inverse dithienylethene derivatives^34^, but in most such cases studied hitherto, the bifurcation arises as a result of sampling a ‘transition-state’-like region of a common excited state (for example, a ππ* state), the potential energy surface of which supports multiple minima.

The current results demonstrate the utility of TRPES with XUV probes for characterizing complex photochemical mechanisms. Comparison with previous experiments^16^ indicates that a systematic examination of the effect of excitation wavelength would be useful. Ideally, this should be coupled to complementary experimental work more sensitive to molecular geometry. For instance, ultrafast electron diffraction and ultrafast X-ray scattering experiments^35–38^ might offer further insights into the dominant conical intersection and, at longer times, reveal fragmentation products from the hot ground-state molecules. We anticipate that future experiments should provide an even deeper understanding of the intriguing ultrafast ring reconfigurations in this photoswitch molecule.

## Methods

### Experiment

The experiment was performed at the Free Electron laser Radiation for Multidisciplinary Investigations (FERMI) FEL facility^39^ at the Low Density Matter (LDM) beamline^40,41^. XUV probe pulses with a photon energy of 18.97 eV were generated at 50 Hz as the fourth harmonic of the seed laser wavelength at 261.4 nm. The UV pump pulses with a central wavelength of 200.6 nm and a bandwidth of 0.6 nm (6.18 ± 0.02 eV) were generated as the fourth harmonic of a Ti:sapphire laser with a sequence of three barium borate crystals. For the data presented here, the pump–probe delay was scanned between −500 and 1,000 fs with a step size of 25 and 50 fs near and far from the pump–probe overlap, respectively. At each delay point, 3,000 shots were acquired. For every second shot, the UV pulse was blocked by inhibiting the trigger of the Ti:sapphire amplifier. Shots without UV excitation pulses present were used to subtract static features in the signal and for additional normalization (Supplementary Section 1.4). The pump–probe instrument response function was obtained by monitoring the UV-induced ionization of helium atoms following resonant 1*s* → 4*p* excitation at 23.72 eV (fifth harmonic of the seed laser), which yielded an instrument response function of 186 ± 28 fs (full-width at half-maximum), as shown in Supplementary Fig. 3 and Supplementary Table 1.

QC was synthesized by adding recrystallized Michler’s ketone (0.35 wt%; 98%, Sigma-Aldrich) to NBD (97%, Alfa Aesar) and irradiating the mixture for 48 hours (Thorlabs, M365LP1; 365 nm). The product was distilled, and the irradiation and distillation process repeated. The final product was checked by ^1^H NMR (400 MHz) and showed a 99% photoconversion of NBD to QC, without significant contamination from other products (Supplementary Section 1.3 for further details). The molecules were delivered into an ultrahigh vacuum chamber as a supersonic molecular beam using a pulsed Even–Lavie valve without further heating and with helium at a backing pressure of 6 bar as a carrier gas.

Photoelectron spectra were measured with a magnetic bottle electron spectrometer described previously^23^. For the data shown here, an effective retardation voltage of 9.7 V was used to increase the resolution in the BE region of interest containing the excited states as well as the lowest-BE peak of ground-state QC. The conversion from time of flight to kinetic energy was calibrated by measuring the helium 1*s* photoline at 28.46 eV (sixth harmonic of the seed laser) and 33.20 eV (seventh harmonic) photon energy. Weaker photoelectron signals corresponding to ionization with the third harmonic of the FEL tuned to the fourth, fifth, sixth and seventh harmonic were also included in the calibration. For the conversion to BE values, the photoelectron kinetic energy was subtracted from the central FEL photon energy. By comparing the XUV photoelectron spectrum of QC measured here with a high-resolution spectrum reported in the literature^16^ (Supplementary Fig. 4), we estimate the kinetic energy resolution to be δE/E ≈ 0.03 (that is, ±0.3–0.5 eV) in the kinetic energy range shown in Fig. 2.

The statistical uncertainty of the experimental signal was estimated using a standard bootstrapping analysis. The ~40,000 single-shot digitizer traces that were analysed to obtain Fig. 2 were randomly resampled with replacement 150 times. The conversion from time of flight to electron kinetic energy (including Jacobian correction) was performed on each bootstrap dataset separately. The mean and standard deviation of these 150 datasets define the values and the corresponding error bars shown in Fig. 2b–e. They were checked for convergence by repeating the bootstrapping analysis a second time and comparing the results of both procedures.

### Calculations

Electronic structure calculations for the neutral species were performed using RMS-CASPT2 (ref. ^42^) based on SA(9)-CASSCF(2,6) with the 6-31G(*d*) basis augmented by additional diffuse functions (8*s*8*p* contracted to 1*s*1*p*) centred on the bridging CH_2_ fragment. The active space contained two valence, one 3*s* Rydberg and three 3*p* Rydberg orbitals (shown in Extended Data Fig. 5). No 3*d* orbitals were included in this study, as their presence was not seen in the experimental data. The lowest six (S_0_–S_5_) states were deemed sufficient to describe the dynamics observed, though state averaging over the first nine roots returned by the calculations was required for convergence. All nine states were active in the simulations; however, only a very small fraction of population ever appeared in the states S_6_ and above during the dynamics. We note that this approach fails to capture well the second valence excited state of NBD^4^, but as discussed in the Supplementary Information, this omission is judged to have little impact on the dynamics initiated by photoexciting QC. Electronic structure calculations for the molecular ion were performed with the same method and basis, although with one electron removed, that is, RMS-CASPT2/SA(6)-CASSCF(1,6), using the neutral orbitals as an initial guess. This accounts for all ionizations from the active electron, including the D_0_ and D_1_ ionic states of interest (further details in Supplementary Information). The absence of Rydberg states in the ion wavefunction leads to lower relative energies in the ion than the neutral, leading to a bias towards lower *E*^IP^ values (Supplementary Information). All calculations included an imaginary shift of 0.5*E*_h_, in units of hartree E_h_=27.2114 eV, to remove intruder states and were performed in a development branch of OpenMolcas (ref. ^43^) implementing gradients for RMS-CASPT2 (ref. ^44^).

The potential energy curves in Fig. 3 are calculated along a LIIC that connects the ground-state equilibrium geometry of QC to the S_1_/S_0_ MECI, and then along a LIIC from this MECI geometry to the ground-state equilibrium geometry of NBD. The C_4_-ring geometry at the MECI is rhombic^4^^,^^18^ and does not lie directly between the equilibrium geometries of the two isomers, as shown in Extended Data Fig. 3. Since this is an essentially barrierless process, there is no obvious conceptual advantage in considering a minimum energy pathway over the LIICs.

Non-adiabatic trajectories were run using the surface-hopping method with a locally modified version of SHARC v.2.1 (ref. ^45^). Couplings were calculated using the local-diabatic approach. Limited numerical issues with the stability of the gradients were mitigated by a variable time step for the integration (Supplementary Section 2.3 for details). The surface-hopping simulations were performed using an electronic time step 1/25 times smaller than the nuclear time step, and used the energy-based decoherence correction with the standard *C* = 0.1*E*_h_.

The initial conditions were selected from a Wigner-sampled ground-state QC geometry using the delta-pulse approximation^46^ inside a 5.75 ± 0.07 eV window, chosen to overlap well with the calculated partial absorptions to the 3*p* Rydberg states of QC (Extended Data Fig. 7). Trajectories were categorized into those excited into S_2_ (121 trajectories, 45%) and those excited into S_3_ and S_4_ (146 trajectories, 55%). Strictly, one-photon excitation S_0_ →S_2_ is forbidden at the equilibrium geometry, and the excitation is therefore attributable to the Herzberg–Teller effect with the transition gaining strength from the mixing of valence character into the S_2_ state. Inspection showed that within the envelope of the Wigner sampling, the character of the states remained separated, with the S_2_ state corresponding to the 3*p*_*x*_/V configuration, whereas the S_3_ and S_4_ states each involved a mixture of excitations to the 3*p*_*y*_ and 3*p*_*z*_ orbitals. This separation is confirmed by the distinctly different, excitation-character-dependent dynamics displayed by the respective sets of trajectories. A very small sample (5 trajectories) was excited to the S_1_ (3*s*) state at this energy. These are not considered further in the current analysis.

For the analysis, the trajectories were sorted according to initial state, allowing resolution of the dynamics into state-specific components. A small portion (7% of the total) of trajectories undergo a retro-Diels–Alder reaction from NBD-like geometries after passing through the MECI geometry, yielding C_5_H_6_ and C_2_H_2_ fragments, but these were excluded from the analysis as the selected active space cannot describe the fragmentation pathway accurately. Molecular geometries were differentiated by the average carbon–carbon distance *r*_CC_ = (*r*_12_ + *r*_34_)/2 (atom numbers shown in Fig. 1), with QC-like geometries defined to have *r*_CC_ < 2.0 Å and NBD-like geometries defined to have 2.0 Å < *r*_CC_ < 3.5 Å.

Photoionization cross-sections and energies were calculated by selecting a random time point in each 5 fs period within each trajectory. The squared Dyson norm^47^ was calculated between the active state in the surface hopping and all ionic states, and used as an approximation to the photoionization cross-section. The method yields qualitatively lower cross-sections for valence states than for Rydberg states when compared to experiment, so for display purposes, the intensities in Fig. 4c with BE > 3 eV have been multiplied by a factor of three (further discussion in Supplementary Section 2.3). The difference in vertical ionization potential between QC and NBD is roughly 0.3 eV with E^IP^_QC_ = 7.67/8.4 eV and E^IP^_NBD_ = 8.02/8.67 eV (first value from present vertical calculations in Table 1 and second value from experiment (ref. ^16^), respectively).

## Online content

Any methods, additional references, Nature Portfolio reporting summaries, source data, extended data, supplementary information, acknowledgements, peer review information; details of author contributions and competing interests; and statements of data and code availability are available at 10.1038/s41557-023-01420-w.

### Supplementary information

Supplementary Information Supplementary Information with further details on the experiment and calculations is available.

### Source data

Source Data Fig. 1 QC, MECI and NBD coordinates in .xyz format (in angstroms).

Source Data Fig. 2 Experimental photoelectron difference spectra.

Source Data Fig. 3 Absolute energies in hartrees; the first six columns are S_0_ to S_5_, and the final two columns are D_0_ and D_1_, from left to right respectively.

Source Data Fig. 4 ASCII data.

**Extended Data Fig. 1:**
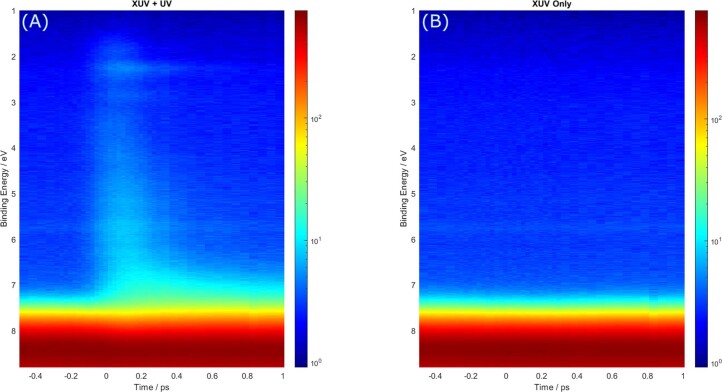
Time-dependent photoelectron spectra of QC with and without pump pulse. During the pump-probe scans, the UV laser was present only for every second FEL shot, such that data with and without pump pulse were recorded in parallel. All data presented in Fig. 2 of the main text are shown as a difference signal of all the accumulated ‘pump on’ (**a**) minus ‘pump off’ **(b**) spectra at each delay point. Note the log scale for the color map in the above spectra to show the weak pump-probe signal in the non-subtracted spectra. Source data

Source Data Extended Data Fig. 1 Photoelectron spectra with and without pump pulse.

**Extended Data Fig. 2:**
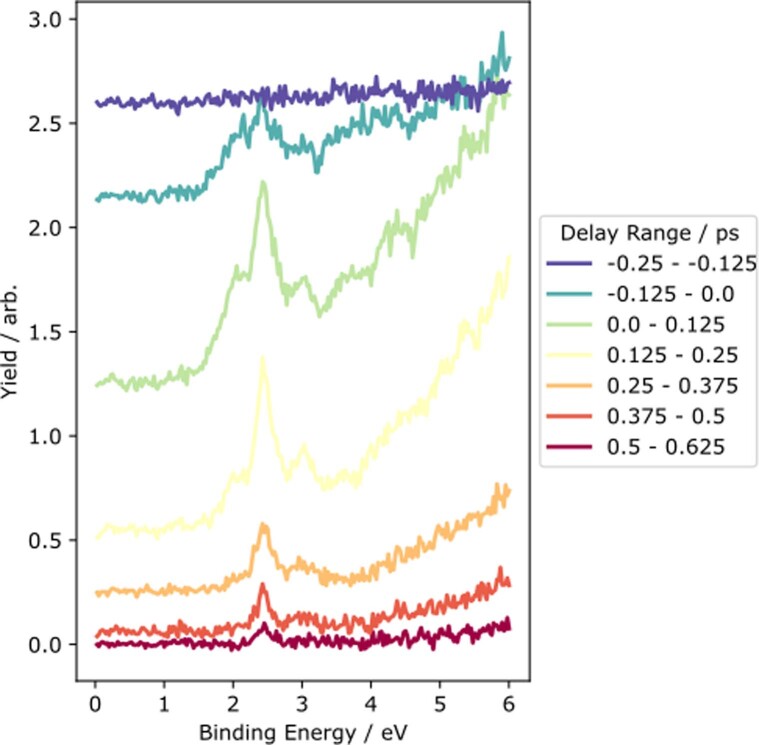
Time-dependent photoelectron difference spectra of QC in the excited-state region at different pump-probe delays. Same data as shown in Fig. 2b but presented in finer delay steps and over a larger delay range. Source data

Source Data Extended Data Fig. 2 Time-dependent photoelectron difference spectra.

**Extended Data Fig. 3:**
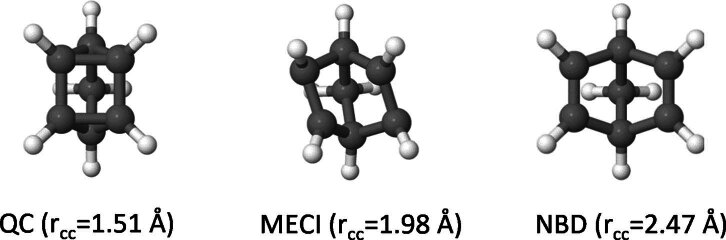
Optimized geometries. The molecular geometries for QC, NBD, and the MECI, optimized using RMS(9)-CASPT2(2,6)/6-31 G* + D level theory. The geometries are shown looking down the z-axis (as shown in Fig. 1 in the main text). Note the rhombic nature of the MECI geometry, which does not lie on the totally symmetric displacement between the two equilibrium geometries of QC and NBD. Source data

Source Data Extended Data Fig. 3 QC, MECI and NBD coordinates in .xyz format (in angstroms).

**Extended Data Fig. 4:**
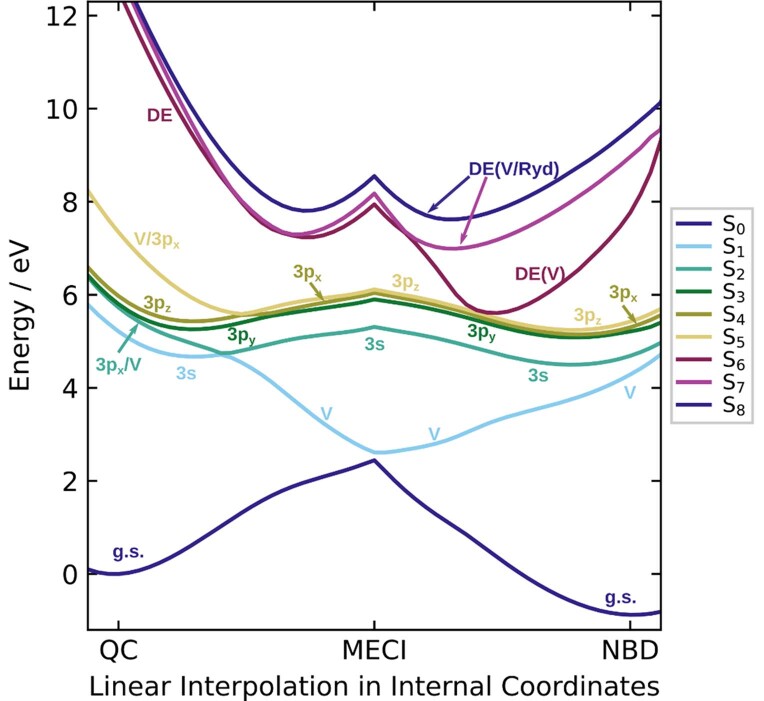
State characters. LIIC for all electronic states considered in the RMS(9)-CASPT2(2,6)/6-31 G* + D model, with approximate character labelled (color coded). Each state is labelled by its principal character (for example, 3p_y_ indicates a state of mostly HOMO **→** 3p_y_ character). DE indicates a doubly excited state, with the character of the orbital(s) excited into in parentheses. Source data

**Extended Data Fig. 5:**
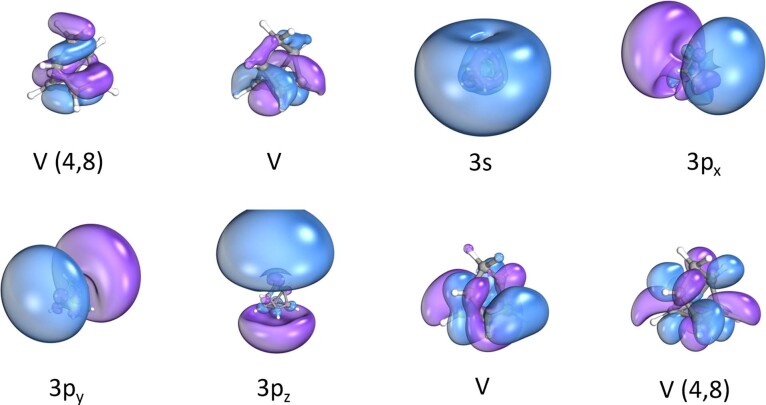
Active space orbitals calculated at QC. The active space consists of 2 electrons in a space of 2 valence and 4 Rydberg orbitals. Extending the active space to include one additional pair of virtual and occupied orbitals creates the (4,8) active space of Valentini *et al*. (Valentini et al., 2020). The two orbitals that extend the (2,6) active space are identified by a (4,8) in their caption. The isosurface cutoff value has been adjusted such that 80% of the total density is shown for each orbital, and Rydberg orbitals are shown zoomed out to fully render their spatial extent. A rendering of the molecule is included in each frame.

Source Data Extended Data Fig. 4 Absolute energies in hartrees.

**Extended Data Fig. 6:**
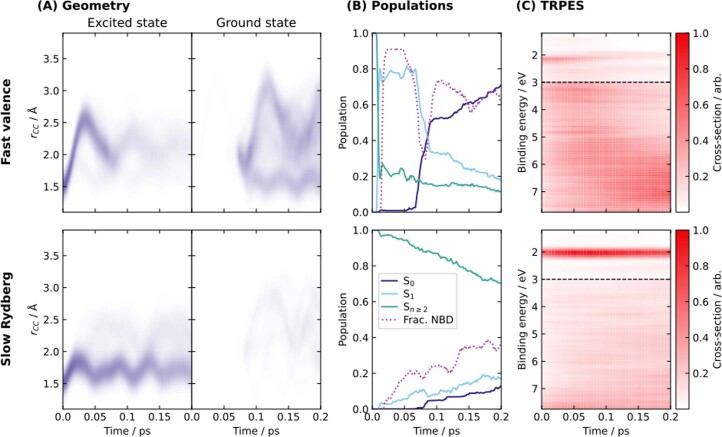
Early-time dynamics from simulations. The same data as shown in Fig. 4 in the main text but zoomed in on the first 200 fs. **a**, False-colour maps for average carbon–carbon distance r_CC_ = (r_12_ + r_34_)/2 versus time. **b**, Populations of the electronic states and fraction of molecules with NBD-like geometry (defined as r_CC_ > 2.0 A) as a function of time. c, Simulated TRPES data, convoluted by Gaussian functions with full-width at half-maximum of 0.1 eV and 94 fs. Signals with BE > 3 eV (below the horizontal dashed line) have been multiplied by a factor of three prior to display. Source data

Source Data Extended Data Fig. 6 ASCII data.

**Extended Data Fig. 7:**
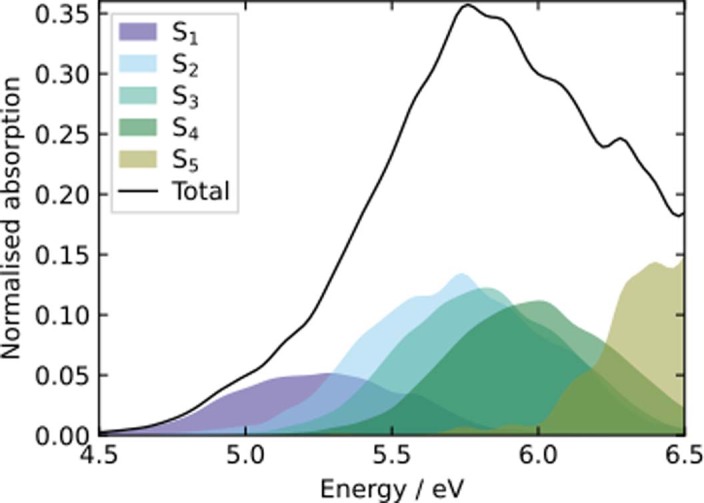
Calculated absorption spectrum. Wigner sampled absorption spectrum of QC using 10000 initial conditions broadened by a Gaussian (FWHM = 0.1 eV). Calculated using geometries and energies at the RMS(9)-CASPT2(2,6)/6-31 G* + D level of theory. Source data

Source Data Extended Data Fig. 7 Cross-sections. Headers of columns indicate data in the column. The 1, 2, 3 and so on indicate the excitation cross-section into states S_0_, S_1_, S_2_ and so on, respectively. Total is the total excitation cross-section.

## Data Availability

Data generated or analysed during this study are included in this Article (and its Supplementary Information). Source data are provided with this paper.

## Code availability

The analysis codes used to generate the data presented in this study are available from the corresponding authors upon reasonable request.

## References

[1] Pianowski, Z. L. *Molecular Photoswitches: Chemistry, Properties, and Applications* Vols 1 and 2 (Wiley, 2022).

[2] Franz E (2022). Tunable energy release in a reversible molecular solar thermal system. ACS Catal..

[3] Wang Z, Hölzel H, Moth-Poulsen K (2022). Status and challenges for molecular solar thermal energy storage system based devices. Chem. Soc. Rev..

[4] Antol I (2013). Photodeactivation paths in norbornadiene. J. Comput. Chem..

[5] Jorner K (2017). Unraveling factors leading to efficient norbornadiene–quadricyclane molecular solar-thermal energy storage systems. J. Mater. Chem. A.

[6] Dreos A (2018). Liquid norbornadiene photoswitches for solar energy storage. Adv. Energy Mater..

[7] Orrego-Hernández J, Dreos A, Moth-Poulsen K (2020). Engineering of norbornadiene/quadricyclane photoswitches for molecular solar thermal energy storage applications. Acc. Chem. Res..

[8] Alex W (2022). Solar energy storage: competition between delocalized charge transfer and localized excited states in the norbornadiene to quadricyclane photoisomerization. J. Am. Chem. Soc..

[9] Coppola F, Nucci M, Marazzi M, Rocca D, Pastore M (2023). Norbornadiene/quadricyclane system in the spotlight: the role of Rydberg states and dynamic electronic correlation in a solar-thermal building block. ChemPhotoChem.

[10] Van Ingen JWF, Van Tieghem CHC, Cramer WA (1970). Radiation-induced isomerization of [2,2,1] bicycloheptadiene (norbornadiene) to [2,2,1,0^2,6^,0^3,5^] quadricycloheptane (quadricyclene) in cyclohexane solutions. J. Chem. Phys..

[11] Jones G, Chiang S-H, Becker WG, Greenberg DP (1980). Structure-reactivity factors for exciplex isomerization of quadricyclene and related compounds. J. Chem. Soc., Chem. Commun..

[12] Jones G, Chiang SH, Becker WG, Welch JA (1982). Photosensitization of quadricyclene isomerization by electron acceptors. A short-circuit nonradiative decay mechanism for electron donor-acceptor quenching in polar media. J. Phys. Chem..

[13] Hillers-Bendtsen AE, Iuel Lunøe Dünweber PG, Olsen LH, Mikkelsen KV (2022). Prospects of improving molecular solar energy storage of the norbornadiene/quadricyclane system through bridgehead modifications. J. Phys. Chem. A.

[14] Hoffmann R (1971). Interaction of orbitals through space and through bonds. Acc. Chem. Res..

[15] Fuß, W., Kuttan Pushpa, K., Schmid, W. E. & Trushin, S. A. Ultrafast [2 + 2]-cycloaddition in norbornadiene. *Photochem. Photobiol. Sci.* **1**, 60–66 (2002). 10.1039/b107442c 12659150

[16] Rudakov F, Weber PM (2012). Ultrafast structural and isomerization dynamics in the Rydberg-exited quadricyclane:norbornadiene system. J. Chem. Phys..

[17] Palmer MH (2020). High-level studies of the ionic states of norbornadiene and quadricyclane, including analysis of new experimental photoelectron spectra by configuration interaction and coupled cluster calculations. J. Chem. Phys..

[18] Valentini A, van den Wildenberg S, Remacle F (2020). Selective bond formation triggered by short optical pulses: quantum dynamics of a four-center ring closure. Phys. Chem. Chem. Phys..

[19] Hernández, F. J., Cox, J. M., Li, J., Crespo-Otero, R. & Lopez, S. A. Multiconfigurational calculations and photodynamics describe norbornadiene photochemistry. *J. Org. Chem*. **88**, 5311–5320 (2023). 10.1021/acs.joc.2c02758 PMC10629221 37022327

[20] Adachi S, Sato M, Suzuki T (2015). Direct observation of ground-state product formation in a 1,3-cyclohexadiene ring-opening reaction. J. Phys. Chem. Lett..

[21] von Conta A (2018). Conical-intersection dynamics and ground-state chemistry probed by extreme-ultraviolet time-resolved photoelectron spectroscopy. Nat. Commun..

[22] Smith AD (2018). Mapping the complete reaction path of a complex photochemical reaction. Phys. Rev. Lett..

[23] Squibb RJ (2018). Acetylacetone photodynamics at a seeded free-electron laser. Nat. Commun..

[24] Pathak S (2020). Tracking the ultraviolet-induced photochemistry of thiophenone during and after ultrafast ring opening. Nat. Chem..

[25] Palmer MH (2023). High-level studies of the singlet states of quadricyclane, including analysis of a new experimental vacuum ultraviolet absorption spectrum by configuration interaction and density functional calculations. J. Chem. Phys..

[26] Cooper, J. C. et al. Valence-shell electronically excited states of norbornadiene and quadricyclane, *J. Chem. Phys.*, 10.1063/5.0187707 (2024). 10.1063/5.0187707 38349638

[27] Jensen JO (2005). Vibrational frequencies and structural determination of cyanogen isocyanate. J. Mol. Struct. THEOCHEM.

[28] Zhou X, Liu R (1996). Density functional theory study of vibrational spectra. 3. Assignment of fundamental vibrational modes of quadricyclane. Vib. Spectrosc..

[29] Jacovella U (2020). Photo- and collision-induced isomerization of a charge-tagged norbornadiene–quadricyclane system. J. Phys. Chem. Lett..

[30] Robin, M. B. *Higher Excited States of Polyatomic Molecules* Vol. 1 (Academic Press, 1974).

[31] Robin, M. B. *Higher Excited States of Polyatomic Molecules* Vol. 3 (Academic Press, 1985).

[32] Forbes, R. et al. Vacuum ultraviolet excited state dynamics of the smallest ketone: acetone. *J. Phys. Chem. Lett*. **12**, 8541–8547 (2021). 10.1021/acs.jpclett.1c02612 34464141

[33] Weir H, Williams M, Parrish RM, Hohenstein EG, Martínez TJ (2020). Nonadiabatic dynamics of photoexcited *cis*-stilbene using ab initio multiple spawning. J. Phys. Chem. B.

[34] Lietard A (2014). Competitive direct *vs.* indirect photochromism dynamics of constrained inverse dithienylethene molecules. Phys. Chem. Chem. Phys..

[35] Minitti MP (2015). Imaging molecular motion: femtosecond X-ray scattering of an electrocyclic chemical reaction. Phys. Rev. Lett..

[36] Stankus B (2019). Ultrafast X-ray scattering reveals vibrational coherence following Rydberg excitation. Nat. Chem..

[37] Wolf TJA (2019). The photochemical ring-opening of 1,3-cyclohexadiene imaged by ultrafast electron diffraction. Nat. Chem..

[38] Yang J (2020). Simultaneous observation of nuclear and electronic dynamics by ultrafast electron diffraction. Science.

[39] Allaria E (2012). Highly coherent and stable pulses from the FERMI seeded free-electron laser in the extreme ultraviolet. Nat. Photon..

[40] Lyamayev V (2013). A modular end-station for atomic, molecular, and cluster science at the low density matter beamline of FERMI@Elettra. J. Phys. B At. Mol. Opt. Phys..

[41] Svetina C (2015). The Low Density Matter (LDM) beamline at FERMI: optical layout and first commissioning. J. Synchrotron Rad..

[42] Battaglia S, Lindh R (2021). On the role of symmetry in XDW-CASPT2. J. Chem. Phys..

[43] Aquilante F (2020). Modern quantum chemistry with [Open]Molcas. J. Chem. Phys..

[44] Nishimoto Y, Battaglia S, Lindh R (2022). Analytic first-order derivatives of (X)MS, XDW, and RMS variants of the CASPT2 and RASPT2 methods. J. Chem. Theory Comput..

[45] Mai, S., Marquetand, P. & González, L. Nonadiabatic dynamics: the SHARC approach. *WIREs Comput. Mol. Sci*. **8**, e1370 (2018). 10.1002/wcms.1370 PMC6220962 30450129

[46] Barbatti M, Sen K (2016). Effects of different initial condition samplings on photodynamics and spectrum of pyrrole. Int. J. Quantum Chem..

[47] Ruckenbauer M, Mai S, Marquetand P, González L (2016). Revealing deactivation pathways hidden in time-resolved photoelectron spectra. Sci. Rep..

