# Supplementary material for: Ultrafast electronic relaxation pathways of the molecular photoswitch quadricyclane
Source: Nat Chem. 2024 Feb 2;16(4):499–505. doi: 10.1038/s41557-023-01420-w (PMC10997510; doi:10.1038/s41557-023-01420-w)
Supplement: Supplementary file 1 — Supplementary Information with further details on the experiment and calculations is available. [file 41557_2023_1420_MOESM1_ESM.pdf]

nature chemistry

Article https://doi.org/10.1038/s41557-023-01420-w

Ultrafast electronic relaxation pathways of the molecular photoswitch quadricyclane

In the format provided by the authors and unedited

Table of Content

1. Experimental Details 1
   1.1. FEL and UV Beam Parameters 1
   1.2. Magnetic Bottle Spectrometer 6
   1.3. Sample Preparation and Handling 7
   1.4. Experimental data analysis 8
   1.5. Fitting of the time-dependent electron yields 10
2. Computational Details 14
   2.1 Electronic Structure 14
   2.2 Character of states 14
   2.3 Dynamics 20
   2.4 Ionic state and photoelectron signal calculation 21
   2.5 Time dependence of calculations 23
   2.6 Absorption spectrum 25

References 26

1. Experimental Details

1.1. FEL and UV Beam Parameters

The experiment was conducted at the Low Density Matter (LDM) beamline (Svetina et al., 2015) at the *Free-Electron laser Radiation for Multidisciplinary Investigations* (FERMI) at the Elettra Synchrotron facility in Trieste, Italy (Allaria et al., 2012). This seeded FEL provides bright XUV and soft X-ray photons at sub-100 fs pulse durations. The FEL pulses are emitted at harmonics of an input seed laser (itself the 3rd harmonic of the fundamental infrared laser source) that pre-bunches the electron beam in a downstream undulator, triggering coherent emission of XUV light rather than relying on shot-noise to initiate the emission process, as is the case in FELs relying on self-amplified spontaneous emission (SASE). Seeded FELs thus deliver high-intensity narrow-bandwidth XUV pulses. The same laser system that generates the seed laser beam is also used to generate the (UV) pump pulses used in the experiment, thus minimizing shot-to-shot timing jitter between the UV and XUV pulses (Cinquegrana et al., 2021). This setup therefore provides good temporal and spectral resolution that have proven effective in studying the UV-induced ultrafast dynamics of gas-phase molecules via valence photoelectron spectroscopy (Pathak et al., 2020; Squibb et al., 2018; Travnikova et al., 2022). For the experiment reported here, the XUV photon energy was 18.97 eV with a bandwidth of 24 meV (FWHM), selected to be below the ionization energy of the helium carrier gas used for the molecular beam. The UV-pump pulse had a central wavelength of 200.6 nm with a bandwidth of 0.6 nm. The 4th harmonic generation setup for obtaining these pulses was based on an original common-path scheme (Susnjar et al., 2023), providing a very high stability. The beam diameter of the UV and XUV in the interaction region was 80 μm (FWHM) and 40 μm (FWHM), respectively. Both the FEL and the UV laser operated at 50 Hz, with the UV laser being blocked every other shot by inhibiting the trigger of the Ti:Sapphire amplifier to allow for interleaved recording of the "unpumped" spectra for subtraction (see SI Section 1.4).

A Sn filter and a gas attenuator, filled with Ne at 9.2 x 10^{-2} mbar, in the beamline were used to suppress any higher harmonics of the FEL. For the data shown here, the average FEL pulse energy behind the filter and attenuator was ≈70 μJ, as monitored on a single-shot basis via a nitrogen gas-monitor that converts the measured nitrogen ion yield to pulse energy (Zangrando et al., 2015). This yields an estimated XUV pulse energy on target of approximately 15 μJ when accounting for the beamline transmission, which is approximately 22% at a photon energy of 20 eV (Svetina et al., 2015). The XUV pulse intensity was chosen such that it provided the highest possible count rate while minimizing multiphoton ionization, broadening of the photoelectron spectra due to space charge, and saturation of the photoelectron detector.

The XUV beam was focused into the LDM end-station by a custom Kirkpatrick-Baez (KB) active-optic focusing system (Raimondi et al., 2013). The system consists of two grazing-incidence mirrors, the first one focusing the beam in the vertical direction, while the second mirror focuses in the horizontal direction. The curvature of both mirrors is fully tunable since their substrate bending is fully assisted by piezo actuators (Manfredda et al., 2022), such that the focal spot size can be tailored to the experimental requirements by monitoring the spot profile on a (removable) Ce:YAG scintillator mounted inside the LDM end-station. Before the two grazing-incidence mirrors, a pair of (horizontal and vertical) slits spatially filters out spurious tails surrounding the main spot of the incoming beam and to select the desired working areas of the downstream focusing optics.

The pulse energy of the UV pulses was controlled via a $\lambda/2$ waveplate and monitored on a shot-by-shot basis with an energy meter. It was recorded in the shot-by-shot data stream along with the XUV pulse energy and the single-shot electron spectra. To determine the optimal UV pulse energy, photoelectron spectra were measured at a delay of 0.5 ps as a function of UV pulse energy, as shown in Supplementary Figs. 1A and 1B. The resulting electron yield as a function of UV pulse energy for several regions of interest is shown in Supplementary Figs. 1C and 1D. For the data shown in this manuscript, a pulse energy of 10 μJ was chosen to yield a reasonably high excitation fraction while safely remaining in the single-photon excitation regime, as demonstrated by the linearity of the excited-state electron yield as a function of UV pulse energy. For comparison, the scans were repeated with a UV pulse energy of 2 μJ, which resulted in the same observations as reported in the main text but with lower statistical significance (see Supplementary Fig. 1).

The FEL-UV temporal instrument response function was determined by recording the time-dependent ion yield from UV-ionization of electronically excited helium atoms produced by resonant 1s → 4p excitation with the FEL undulator set to the 5th harmonic of the seed, 23.72 eV. The ion yield and fit to the data are shown in Supplementary Fig. 3, yielding a Gaussian instrument response with $\sigma = 79\ fs$ (186 fs FWHM). We note that since this cross-correlation measurement was done at the very beginning of the beamtime, more than 24 hours before the data shown in the main text were recorded, the time zero (corresponding to temporal overlap of the UV and XUV pulses) for the QC scan was determined from the fit of the depletion signal shown in Supplementary Fig. 10. Applying that same time zero to the He scan yields a temporal offset of 83 fs between the two, which we attribute to a temporal drift in the time between the two measurements. We carefully analyzed the QC scan data recorded over several "loops" but did not observe any temporal drifts over the time period of those scans.

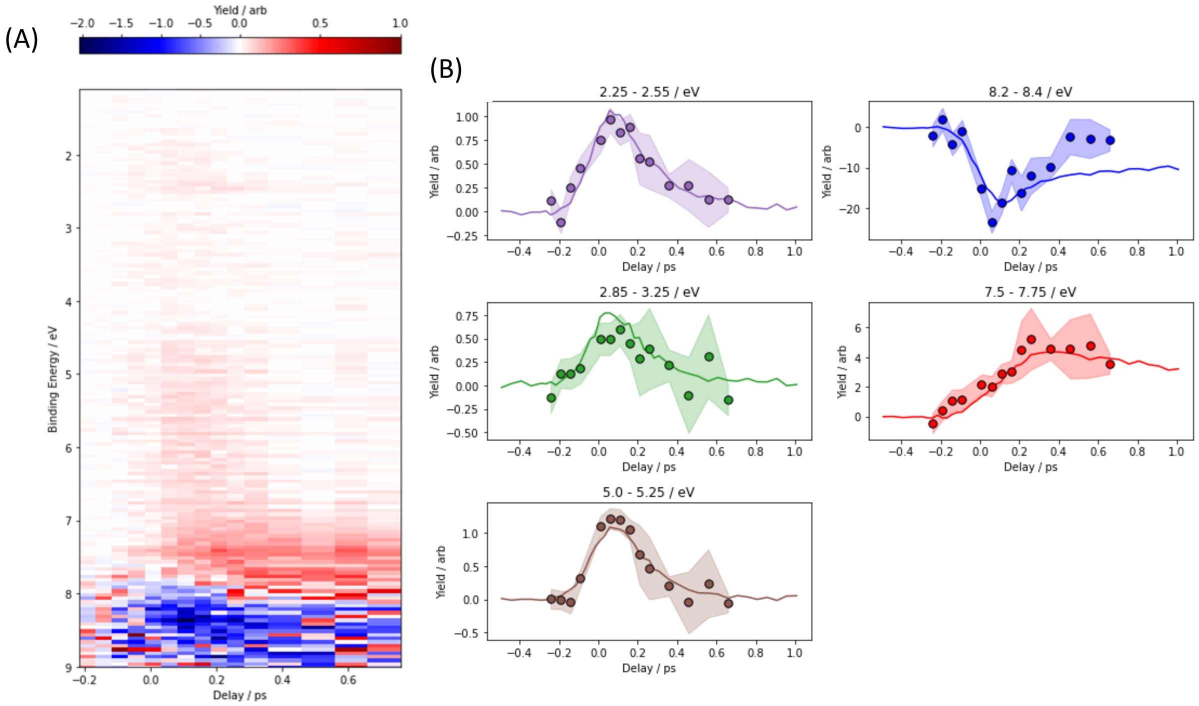

Supplementary Figure 1: Results for lower pump pulse energy. (A) 2-D difference spectrum as shown in Fig. 2A of the main text but with a pump pulse energy of 2 μJ. (B) Lineouts of the 10 μJ data (solid lines) presented in the main text compared to the scaled 2 μJ data (circles). The data and error bands represent the mean value and the 68% confidence interval obtained from a bootstrapping analysis (see Methods).

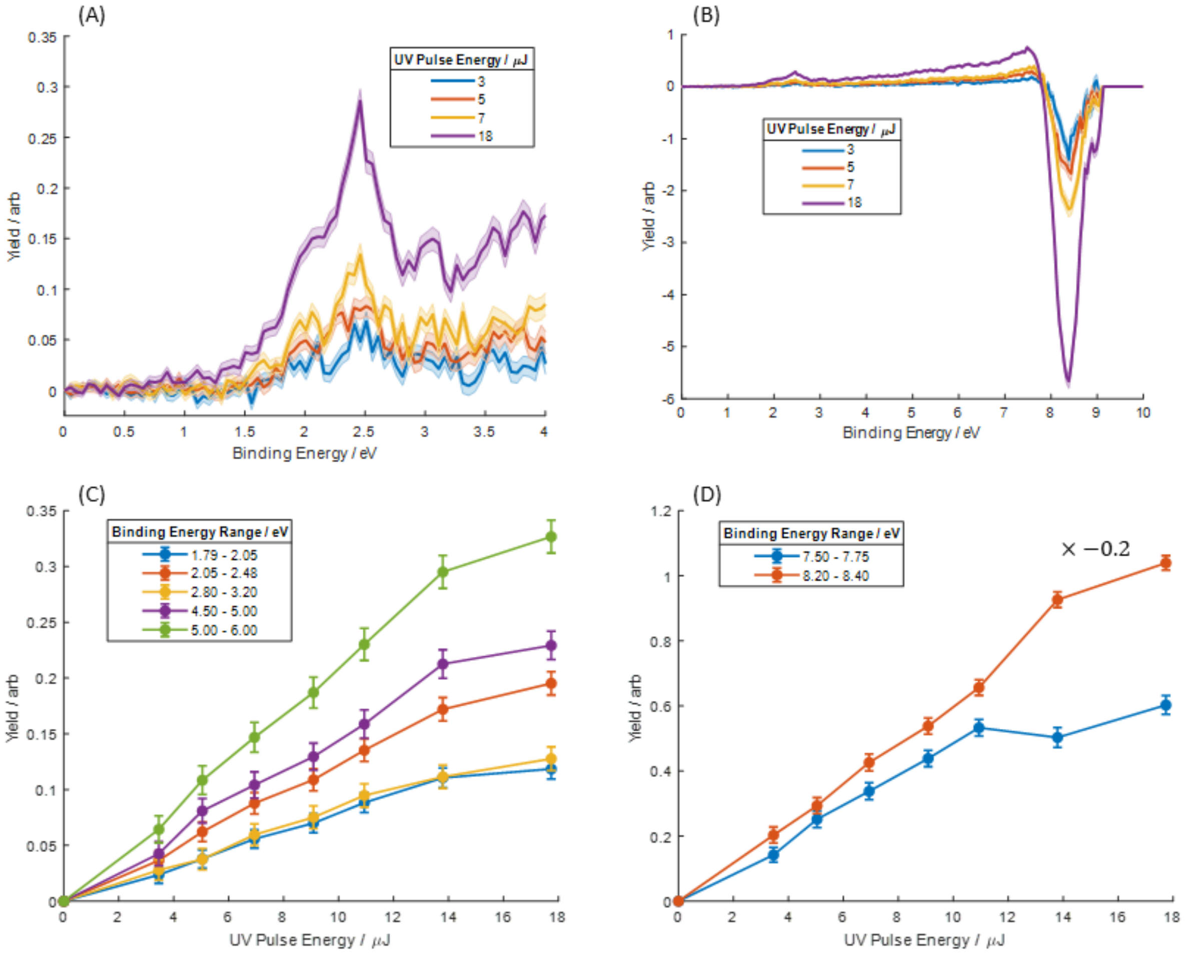

Supplementary Figure 2: Fluence-dependence of the pump-probe signal. (A) Photoelectron spectra of QC in the excited-state region (without subtraction of the "UV-off" signal) at different UV pulse energies at a delay of 0.5 ps. (B) Same as (A) but shown for the entire binding energy range that was recorded (i.e., including the ground-state region) and *with* subtraction of the "UV-off" signal. (C), (D) Integrated electron yield in the indicated binding energy regions as a function of UV pulse energy. The data in the main text were acquired with 10 μJ UV pulse energy. The red data points in (D) were scaled by a factor of -0.2 to fit on the same scale as the blue data points. The data and error bands in (A), (B) and error bars in (C), (D) represent the mean value and the 68% confidence interval obtained from a bootstrapping analysis (see Methods).

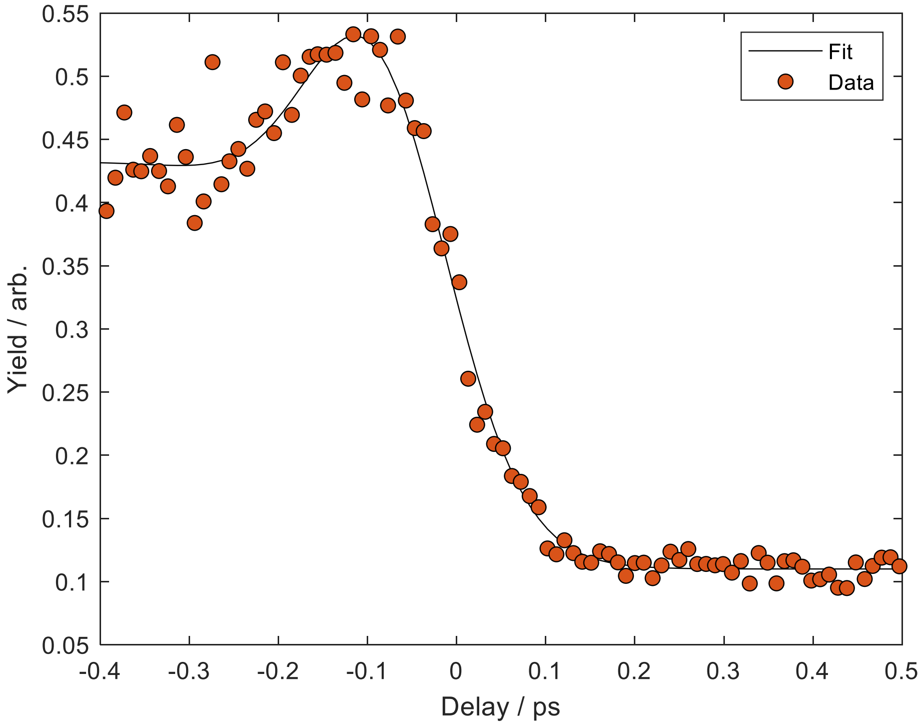

Supplementary Figure 3: Characterization of the pump-probe instrument response function. To estimate the temporal resolution (σ) and determine the delay-stage position corresponding to time overlap (t_0), a supersonic beam of helium atoms was excited at 23.72 eV photon energy (generated by the fifth harmonic of the FEL seed laser) to the 1s → 4p resonance and subsequently ionized by the UV pulse at 200.6 nm. The resulting ion yield was plotted as a function of pump-probe delay. To model the process, a two-component fit was applied to the He^+ signal (see Eq. 1). One component models the (1+1') resonant ionization described above via the convolution of a step function with a Gaussian (i.e., a Gaussian error function), the other models the contribution from a *non-resonant* (1+1') ionization via a pure Gaussian:

$$f(t) = A\left(1 + \mathrm{erf}\left(\frac{t-t_0}{2\sigma}\right)\right) + B\exp\left(-\frac{(t-t_0)^2}{(2\sigma^2)}\right) \qquad (1)$$

Supplementary Table 1: Fit parameters of the least-squares fit shown in Supplementary Fig. 3.

Parameter	Value (95% confidence intervals)
A	-0.161 (0.011) / arb. units
B	0.244 (0.035) / arb. units
σ	79 (12) / fs
t_0	83 (14) / fs

1.2. Magnetic Bottle Spectrometer

The photoelectron spectra were measured using a magnetic bottle electron spectrometer (MBES) with a ≈2-meter flight tube. At the end of the flight tube, electrons were detected by a 40-mm-diameter MCP detector operated at 2200 V bias voltage and with 2400 V applied to the detector anode. The MBES configuration was identical to the one described in Squibb et al. (Squibb et al., 2018), but differs from the one used by Pathak et al. (Pathak et al., 2020), which had a hollow magnet to allow for simultaneous ion detection. The solid-magnet configuration was chosen for the present experiment since it has slightly better (nominal) kinetic energy resolution.

For the data presented here, the solenoid current was set at 0.9 A, and a nominal retardation voltage of 11 V was applied to the drift tube to increase the kinetic energy resolution of the photoelectrons of interest. Combined with a small (1 V) bias voltage on the magnet, this resulted in an effective retardation potential of 9.7 V, according to our kinetic energy calibration. Additional data were also taken at lower and higher retardation voltages, and one example of a spectrum taken without retardation is shown in Supplementary Fig. 4. From this, a kinetic energy resolution of $\frac{\delta E}{E} \sim 0.03$ was estimated for electrons with ≈10 eV kinetic energy. This was estimated by comparing the photoelectron spectrum of QC measured without retardation voltage to a high-resolution spectrum from the literature recorded with synchrotron radiation at a photon energy of 95 eV (Palmer et al., 2020).

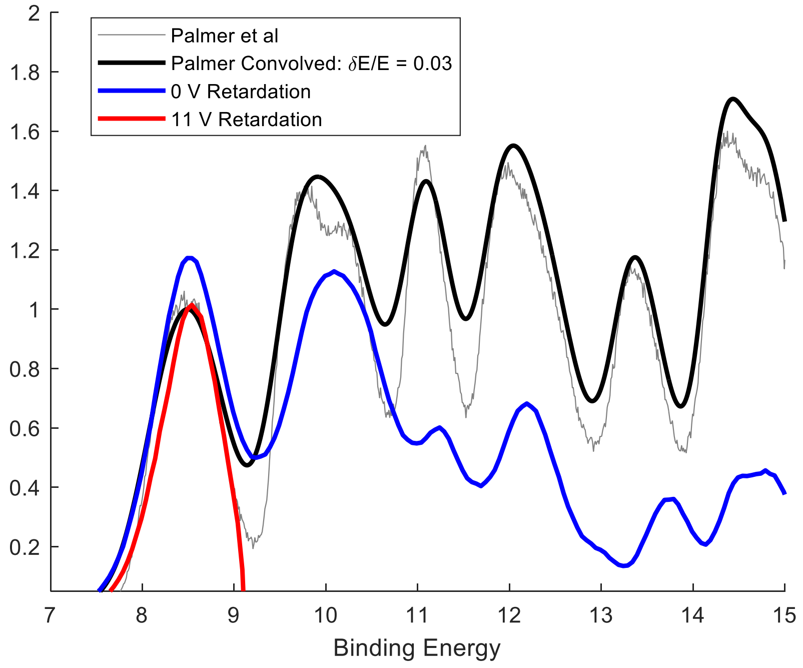

Supplementary Figure 4: Comparison of the measured photoelectron spectra with literature & energy resolution estimate. To estimate the energy resolution of our experiment, the measured QC photoelectron spectrum at 19 eV photon energy *without* retardation applied to the magnetic bottle spectrometer (blue) is compared to a high-resolution photoelectron spectrum (Palmer et al., 2020) at 95 eV photon energy (thin grey line). In order to estimate the energy resolution of the MBES spectrometer, the reference spectrum is convolved with a Gaussian such that it resembles the MBES spectrum. A convolution with a Gaussian of width $\frac{\delta E}{E} \sim 0.03$ yields a satisfactory resemblance (thick black line).

1.3. Sample Preparation and Handling

Quadricyclane (QC) was synthesized by adding recrystallized Michler's ketone (0.35 wt%) (98%, Sigma-Aldrich) to norbornadiene (NBD) (97%, Alfa Aesar) and irradiating the mixture for 48 h (Thorlabs, M365LP1; 365 nm). The product was distilled, and the irradiation and distillation process repeated. The final product was checked by ^1H NMR (400 MHz) and resulted in 99% photoconversion of norbornadiene to quadricyclane, without significant contamination from other products.

The ^1H NMR spectrum of quadricyclane (QC) was measured on a Bruker AVANCE 400WB spectrometer and is shown in Supplementary Fig. 5. The spectrum was recorded at room temperature at 400 MHz and the raw data processed with MestRecC (Cobas & Sardina, 2003). The sample was prepared in $CDCl_3$ (99.8 atom % D, Sigma-Aldrich) and its residual solvent peak used as the reference for the chemical shifts, given in parts per million. ^1H NMR (400 MHz, RT, $CDCl_3$): NBD: δ 1.98 (t, CH_2, bridge, 2H), 3.58 (m, CH, bridgeheads, 2H), 6.76 (m, HC=CH, double bonds, 4H); QC: δ 1.36 (m, CH, bridgeheads, 2H), 1.49 (m, -CH, 4H), 2.02 (t, CH_2, bridge, 2H); $CDCl_3$: δ 7.26 (s); grease: δ 0.07 (s), water: δ 1.54 (s) (Fulmer et al., 2010)

The degree of photoconversion was calculated by using the integrals of all norbornadiene (I_{NBD}) and quadricyclane (I_{QC}) peaks ($[I_{QC} / (I_{QC} + I_{NBD})] \cdot 100\%$). A conversion of 99% from NBD to QC was achieved. The photoelectron spectra of unpumped QC shows no sign of NBD impurity.

During the experiment, the liquid QC sample was kept in a stainless-steel reservoir and introduced into the ultrahigh vacuum chamber as a pulsed molecular beam using an Evan-Lavie valve with a 26 μs opening time and using helium at a backing pressure of 6 bar as carrier gas.

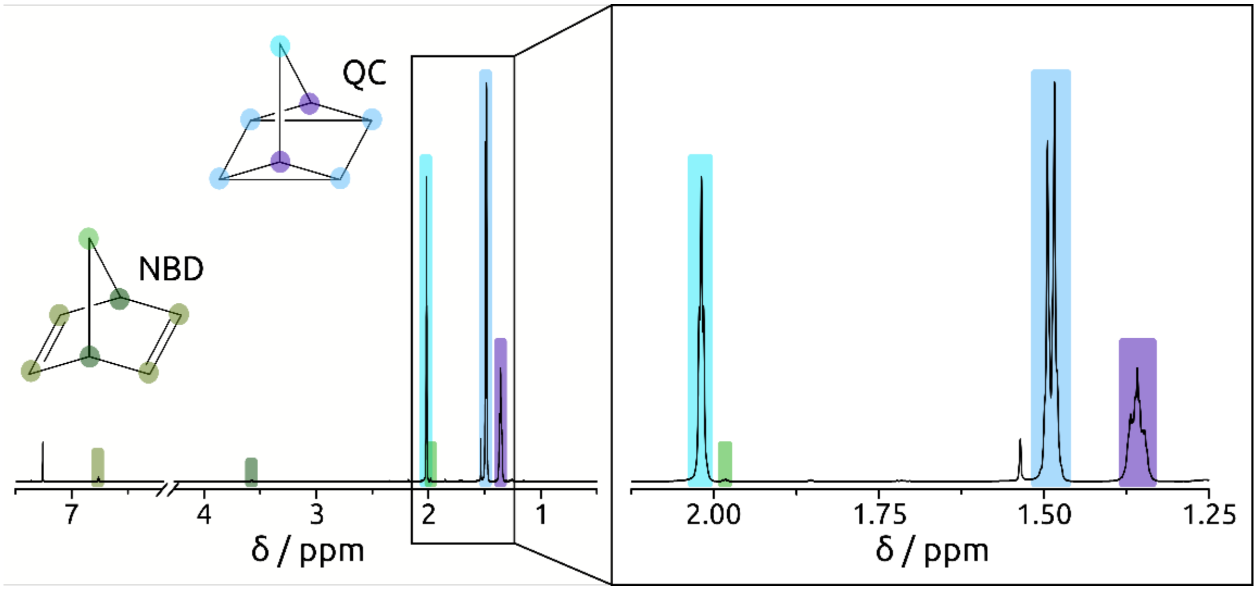

Supplementary Figure 5: ^1H NMR of the quadricyclane sample with color coding of QC and NBD signals.

1.4. Experimental data analysis

As described in the main text, photoelectron spectra were recorded on a single-shot basis. The analog signals from the MCP detector were saved by a 1-GHZ digitizer (CAEN, model VX1751) with 1-ns time bins. FEL pulses are tagged, and their index ("bunch number") is recorded in the data stream for unambiguous event sorting, such as separating the shots where the UV pump was blocked (all odd indices). After sorting events by the delay, the shot-integrated waveforms for a given delay were normalized by the corresponding shot-integrated pulse energies, and the normalized signal from shots without the UV pump pulse was subtracted. To account for any instability beyond pulse energy fluctuations, these values were divided by the total electron yield in the unpumped shots at each delay:

$$S_b(\tau) = \left(\frac{\sum_{i=\text{ even}} Q_{bi}}{\sum_{i=\text{ even}} P_i} - \frac{\sum_{i=\text{ odd}} Q_{bi}}{\sum_{i=\text{ odd}} P_i}\right) / \sum_b \sum_{i=\text{ odd}} Q_{bi} \qquad (2)$$

Here, $S_b(\tau)$ is the processed data at digitizer bin b and delay τ. Q_{bi} and P_i are the analogue signal (in arbitrary analog-to-digital units) and pulse energy (in units of μJ) corresponding to shot i . The pump-probe delay was scanned between -0.5 to 1.0 picoseconds with varying step sizes. Near pump-probe overlap (-0.25 to 0.25 ps), steps of 25 fs were taken, while steps of 50 fs were taken outside of this range. At each delay value, 3000 shots were acquired. This scan range was repeated four times for the data presented in this paper.

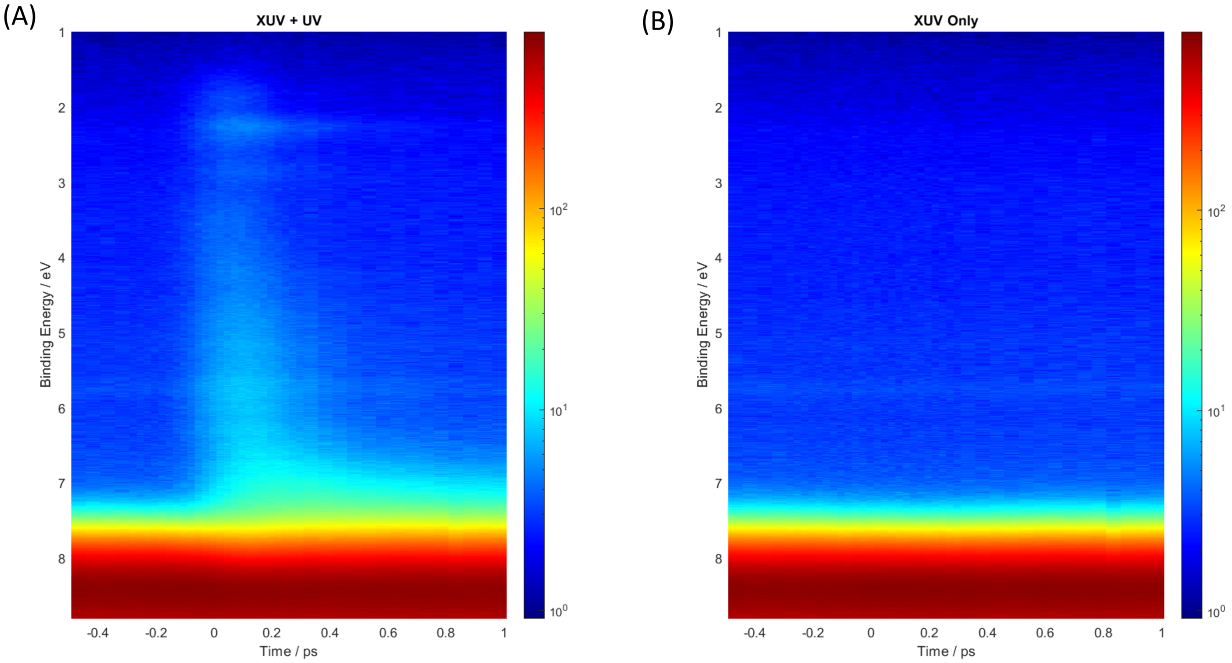

Supplementary Figure 6: Time-dependent photoelectron spectra of QC with and without pump pulse. During the pump-probe scans, the UV laser was present only for every second FEL shot, such that data with and without pump pulse were recorded in parallel. All data presented in the main text are shown as a difference signal of all the accumulated "pump on" (A) minus "pump off" (B) spectra at each delay point. Note the log scale for the color map in the above spectra to show the weak pump-probe signal in the non-subtracted spectra. This figure is the same as Extended Data Fig. 1.

At delay values where the XUV probe precedes the UV pump pulse, subtle features in the difference maps attributed to space-charge broadening and shifting (Zhou et al., 2005) are observed. We assign this to additional space-charge when the UV pulses are present since they create additional photoelectrons by multiphoton ionization. This effect results in a constant difference feature in the region of the ground-state photolines, as shown in Supplementary Fig. 7. Since the space-charge lifetime is on the order of nanoseconds (Zhou et al., 2005), the spurious difference signal due to space charge was removed from the two-dimensional photoelectron spectra by subtracting the difference spectra averaged over the first 5 delay bins (i.e., before time-zero) from each individual delay bin in the 2D map.

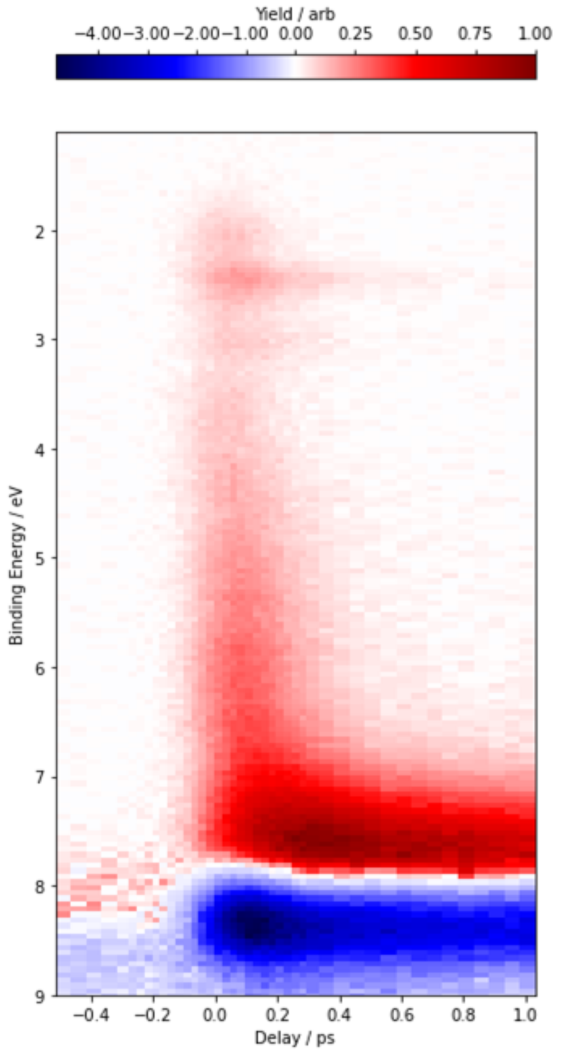

Supplementary Figure 7: Space charge effects. 2-D difference spectrum as shown in Fig. 2A of the main text but without the space-charge correction. The small, persistent difference feature around 8-9 eV BE is attributed to a line broadening and shifting due to space charge.

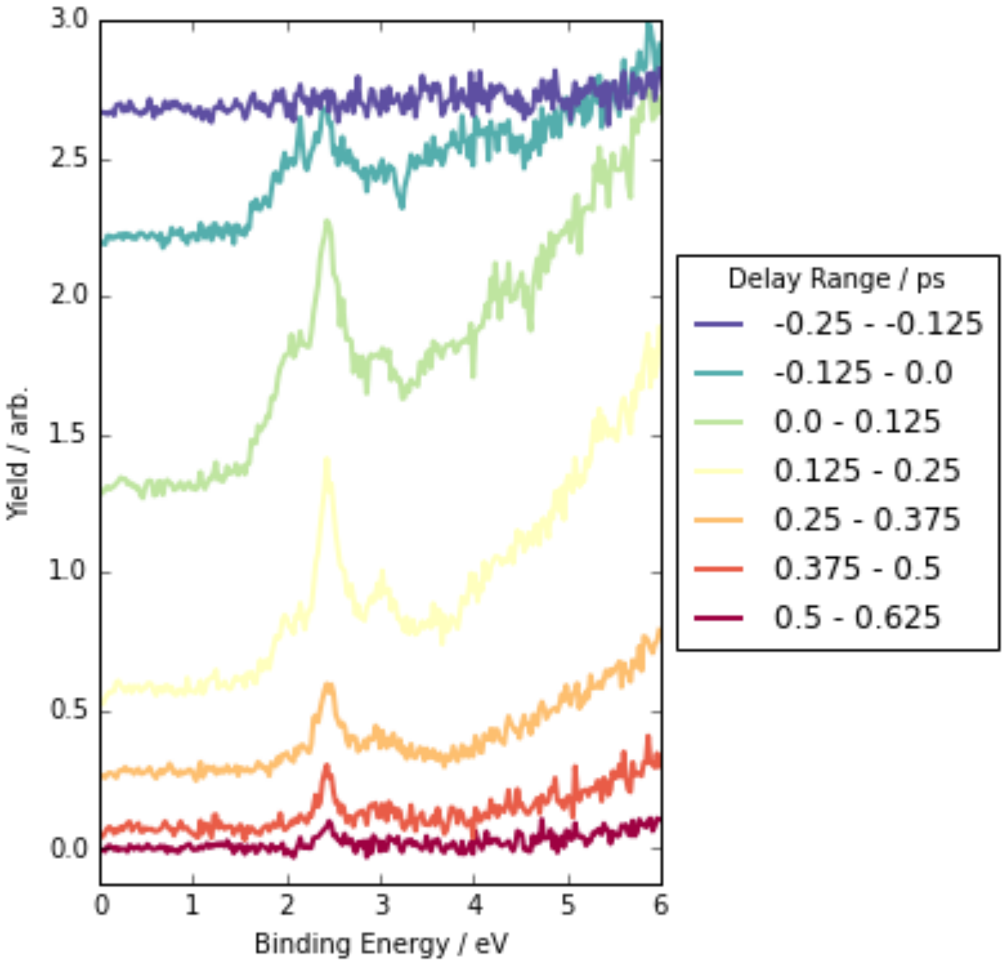

Supplementary Figure 8: Time-dependent photoelectron difference spectra of QC in the excited-state region at different pump-probe delays. This figure is the same as Extended Data Fig. 2.

1.5. Fitting of the time-dependent electron yields

A variety of fit models were applied to describe the time-dependent electron yields, shown, e.g., in Fig. 2(D) and 2(E) of the main text and Supplementary Fig. 9 below, including global fitting routines and various kinetic models. Since the resulting fit parameters depended strongly on the fit model, we opted to show below the results of the most basic model, namely the independent fit of each line-out in the excited-state region with an exponential decay convolved with a Gaussian:

$$f(t) = A\exp\left(\frac{\sigma^2 - \tau(t-t_0)}{\tau^2}\right)\left(1 + \mathrm{erf}\left(\frac{t-t_0}{2\sigma}\right) - \frac{\sigma}{\tau}\right) \quad (3)$$

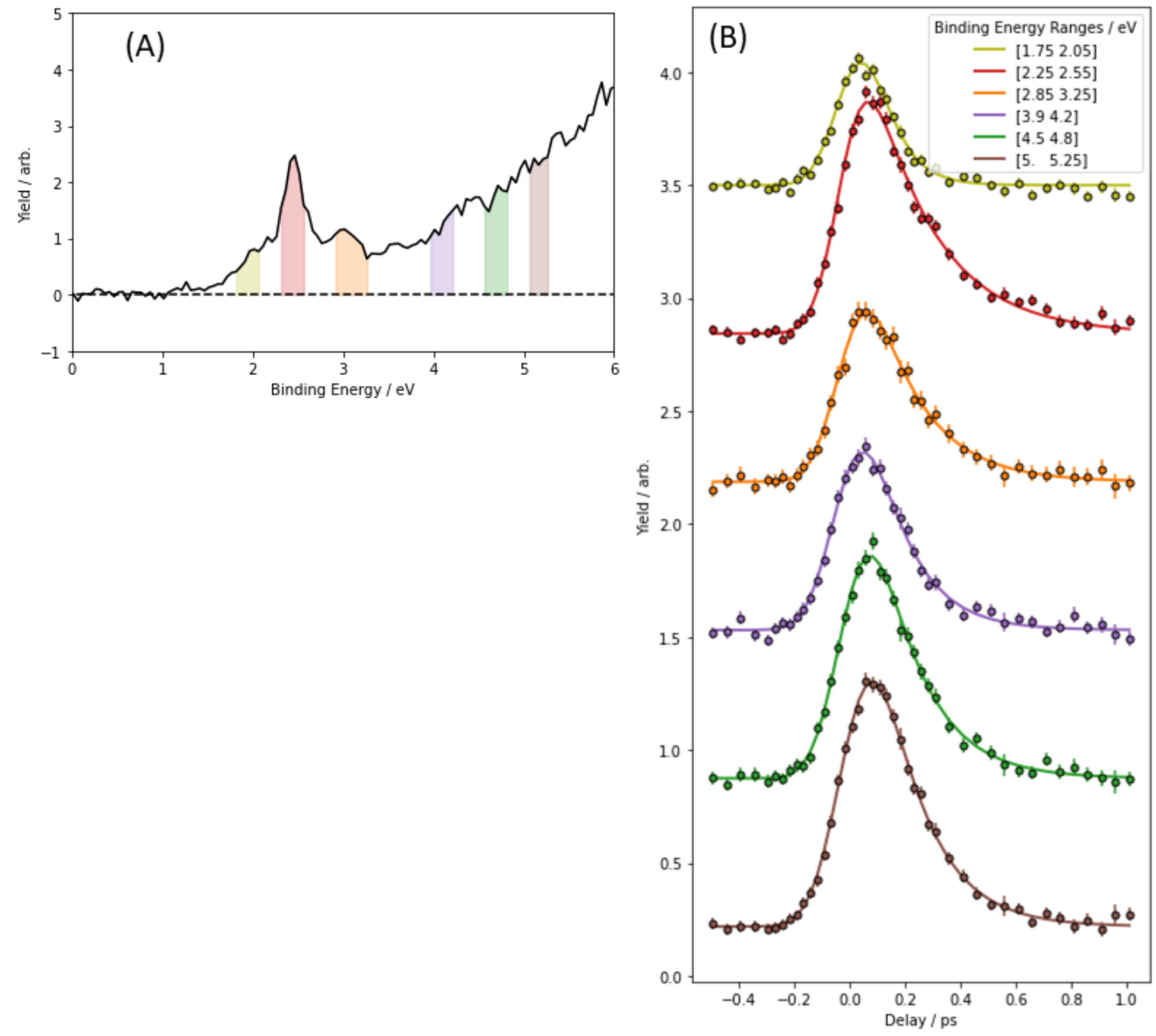

Supplementary Figure 9: Fitting of the photoelectron spectra in the excited-state region. Panel (A) shows the photoelectron spectrum integrated over all delays. The integrated photoelectron yield in the regions marked in panel (A) is fitted with an exponential decay convolved with a Gaussian, see Eq. (3), as shown in panels (B). The data and error bars in (B) represent the mean value of approximately 40,000 single-shot digitizer traces and the 68% confidence interval obtained from a bootstrapping analysis (see Methods). The resulting fit parameters are summarized in Supplementary Table 2.

Supplementary Table 2: Fit parameters of the least-squares fit shown in Supplementary Fig. 9.

Binding Energy / eV	A / arb	τ / fs	σ / fs	t_0 / fs
1.75-2.05	0.85 (0.21)	82 (21)	56 (6)	-15 (13)
2.25-2.55	0.90 (0.05)	228(16)	55 (4)	-26 (5)
2.85-3.25	0.75 (0.10)	182 (29)	58 (7)	-25 (11)
3.90-4.20	0.92 (0.12)	142 (21)	61(6)	-32 (10)
4.50-4.80	1.06 (0.11)	165 (19)	61 (5)	-13 (8)
5.00-5.25	1.23 (0.11)	160 (16)	66 (4)	-7 (7)

Fitting the region of the 3p and 3s Rydberg states with a two-component fit model consisting of *two* exponential decay functions convolved with a Gaussian, as shown in Supplementary Fig. 10, yields a time constant between 240 and 300 fs (see Supplementary Table 3) but requires significant constraints on the fit parameters for the fit to converge.

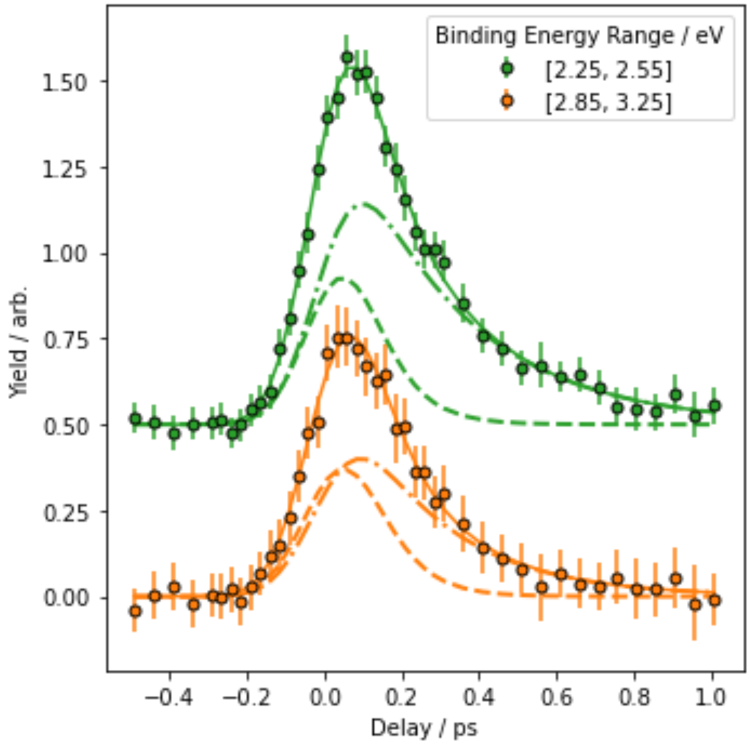

Supplementary Figure 10: Fitting of the photoelectron spectra in the Rydberg-state region with a two-component model. The experimental data is the same as in Supplementary Fig. 9 but fitted with *two* exponential decay functions convolved with a Gaussian. To improve the convergence of the fit, the time constant of the faster decay is fixed to the value obtained from fitting the lowest binding energy interval in Supplementary Fig. 9. The resulting fit parameters for the two-component fit are summarized in Supplementary Table 3.

Supplementary Table 3: Fit parameters of the least-squares fit shown in Supplementary Fig. 10.

**Binding Energy / eV**	**A_1 / arb**	**A_2 / arb**	**τ_1/ fs**	**τ_2/ fs**	**σ/ fs**	**t_0 / fs**
2.25-2.55	632 (43)	364 (31)	82 (fix)	241 (19)	63 (3)	-10 (4)
2.85-3.25	702 (83)	527 (44)	82 (fix)	297 (16)	60 (2)	-11 (3)

The time-dependent electron yield in the ground-state region, shown in Supplementary Fig. 11, displays an enhancement at low BE, which is also fitted by Eq. (3), and a depletion and recovery at higher BE, which is modelled with two contributing terms given in Eq. (4): a prompt decay and an exponential rise, both convolved with the instrument response.

$$f(t) = A\,\mathrm{erfc}\left(\frac{t-2t_0}{2\sigma}\right) + B\left[\left(1+\mathrm{erf}\left(\frac{t-2t_0}{\sqrt{2}\,\sigma}\right)\right) - \exp\left(\frac{\sigma^2-2\tau(t+t0)}{2\tau^2}\right)\mathrm{erfc}\left(\frac{\sigma^2-\tau(t-t_0)}{\sqrt{2}\sigma\tau}\right)\right] \qquad (4)$$

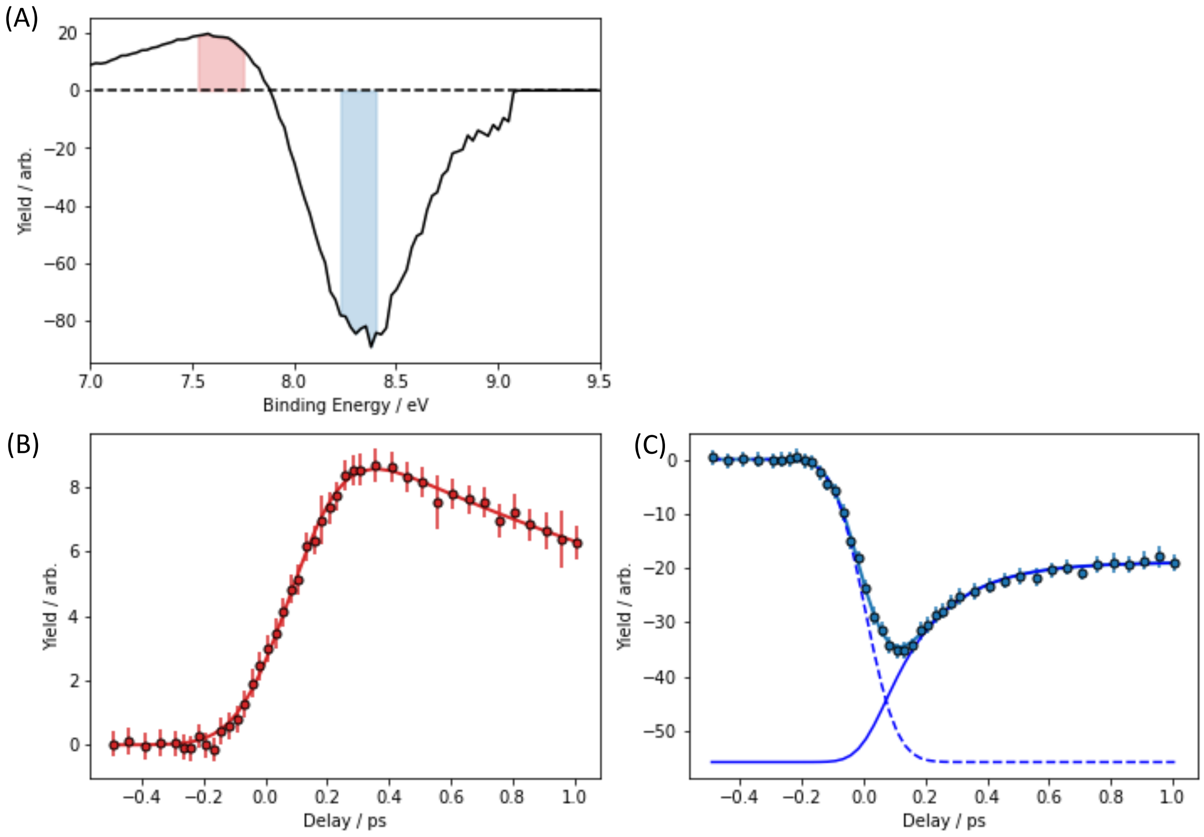

Supplementary Figure 11: Fitting of the photoelectron spectra in the ground-state region. (A) Photoelectron difference spectrum in the BE region 7.0 – 9.5 eV integrated over all delays. (B) The integrated photoelectron yield in the region corresponding to the photoproducts, marked in red in panel (A), is fitted with Eq. (3). (C) The depletion and recovery of the region corresponding to the (cold) QC ground-state, marked in blue in panel (A), is modelled with two contributions given in Eq. (4): a prompt decay (dashed light blue) and an exponential rise (solid blue line), both convolved with the instrument response. The data and error bars in (B), (C) represent the mean value of approximately 40,000 single-shot digitizer traces and the 68% confidence interval obtained from a bootstrapping analysis (see Methods). The resulting fit parameters are summarized in Supplementary Table 4.

Supplementary Table 4: Fit parameters of the least-squares fit shown in Supplementary Fig. 11.

Binding Energy / eV	A / arb.	B / arb.	τ / fs	σ / fs	t_0 / fs
7.5-7.75	5080 (90)	---	1616 (120)	98 (3)	86 (4)
8.2-8.4	27866 (1605)	36780 (2994)	178 (16)	57 (2)	3 (2)

2. Computational Details

2.1 Electronic Structure

QC presents a challenge to electronic structure methods due to the variety of states and the large configuration space explored in the reaction. Supplementary Fig. 12 shows the three molecular geometries discussed in the main text (QC to MECI to NBD). The mean carbon separation r_{cc}, identified as the 'wing-flapping' (opening) motion, increases uniformly from QC to the MECI and then onwards to NBD.

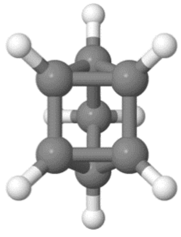	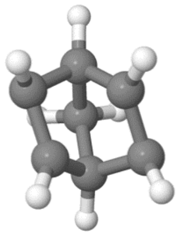	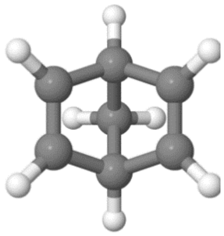
QC (r_{cc}=1.51 Å)	MECI (r_{cc}=1.98 Å)	NBD (r_{cc}=2.47 Å)

Supplementary Figure 12: Optimized geometries. The molecular geometries for QC, NBD, and the MECI, optimized using RMS(9)-CASPT2(2,6)/6-31G*+D level theory. The geometries are shown looking down the z-axis (as shown in Figure 1 in the main manuscript). Note the rhombic nature of the MECI geometry, which does not lie on the totally symmetric displacement between the two equilibrium geometries of QC and NBD. This figure is the same as Extended Data Fig. 3.

For the dynamics simulations, a viable electronic structure model was devised which captures the key features of the molecular system while remaining computationally feasible. The method chosen is rotated multi-state (RMS-CASPT2), a multi-state perturbative correction to the CASSCF method recently developed by Battaglia and Lindh (Battaglia & Lindh, 2021). This constitutes an effective compromise between the well-known multi-state (MS-CASPT2) and extended multi-state (XMS-CASPT2) variants. The method accounts for the multiconfigurational character of the excited states and recovers a sizeable amount of the dynamic correlation in the system. The key aspects to consider are the basis and the active space, discussed in the following.

We also point out that the S_n numbering of the adiabatic electronic states is linked to the electronic structure model used. For example, the V/3p_x state in QC (at 7.53 eV) is not the true experimental S_5 – which is likely a 3d state – but is needed within the confines of our model. The model includes all electronic states relevant to the dynamics, but excludes states high in energy that are not seen in the dynamics, for example the 3d Rydberg states and some valence states in NBD (see later discussion).

2.2 Character of states

To justify the electronic structure model, we make comparisons of potential energy curves (PECs) along the same set of linear interpolation in internal coordinates (LIICs) as shown in Fig. 3 in the main text. The PECs join QC to the MECI, and then onwards to the NBD, using the geometries shown in Supplementary

Fig. 12 which have been optimized at the RMS-CASPT2(2,6) level of theory. We use the same coordinates in all PEC comparisons.

Firstly, we explore the character of the states across the LIIC. The method used calculated 9 roots, only 6 of which are seen with significant population in the dynamics. The primary reason for this is the inclusion of a state with double excitation character, the inclusion of which is important for a correct description of the conical intersection. Supplementary Fig. 13 shows the PECs with the dominant state characters indicated along the LIIC for all 9 roots. As the energies plotted come from adiabatic states, the exact nature of the states changes as a function of the geometry, and so we have indicated only the approximate character.

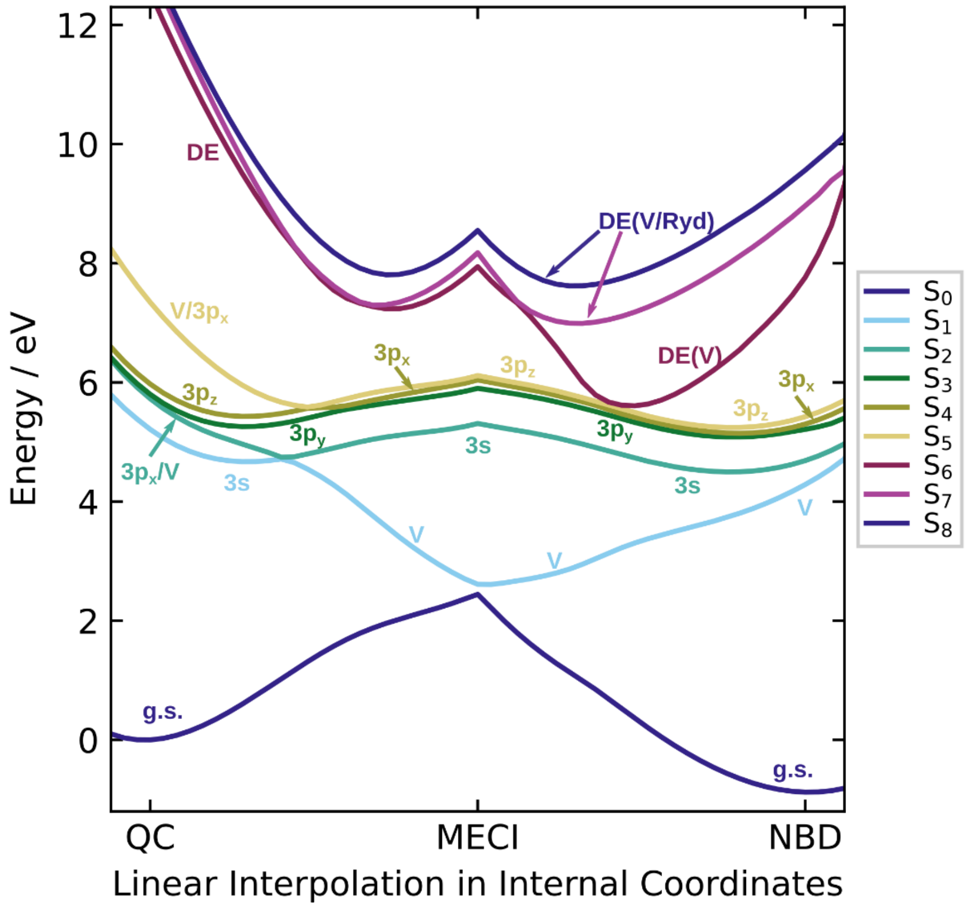

Supplementary Figure 13: State characters. LIIC for all states considered in the RMS(9)-CASPT2(2,6)/6-31G*+D model, with approximate character labelled (color coded). Each state is labelled by its principal character (e.g., $3p_y$ indicates a state of mostly HOMO →$3p_y$ character). DE indicates a doubly excited state, with the character of the orbital(s) excited into in parentheses. In all comparisons that follow hereafter, we show only the lowest 7 adiabatic states (6 which are important to the dynamics and one additional state for reference). This figure is the same as Extended Data Fig. 4.

The comparison between CASSCF and RMS-CASPT2 PECs is shown in Supplementary Fig. 14. The dashed CASSCF curves are clearly quite different to the solid RMS-CASPT2 curves, especially in the Rydberg manifold. This is because diffuse Rydberg orbitals require less dynamic correlation than valence orbitals,

and so in CASSCF, which has little dynamic correlation, the valence states are artificially raised in comparison to Rydberg states. RMS-CASPT2, which includes more correlation than CASSCF, therefore provides more balanced and thus accurate energies of the Rydberg states in relation to the valence states.

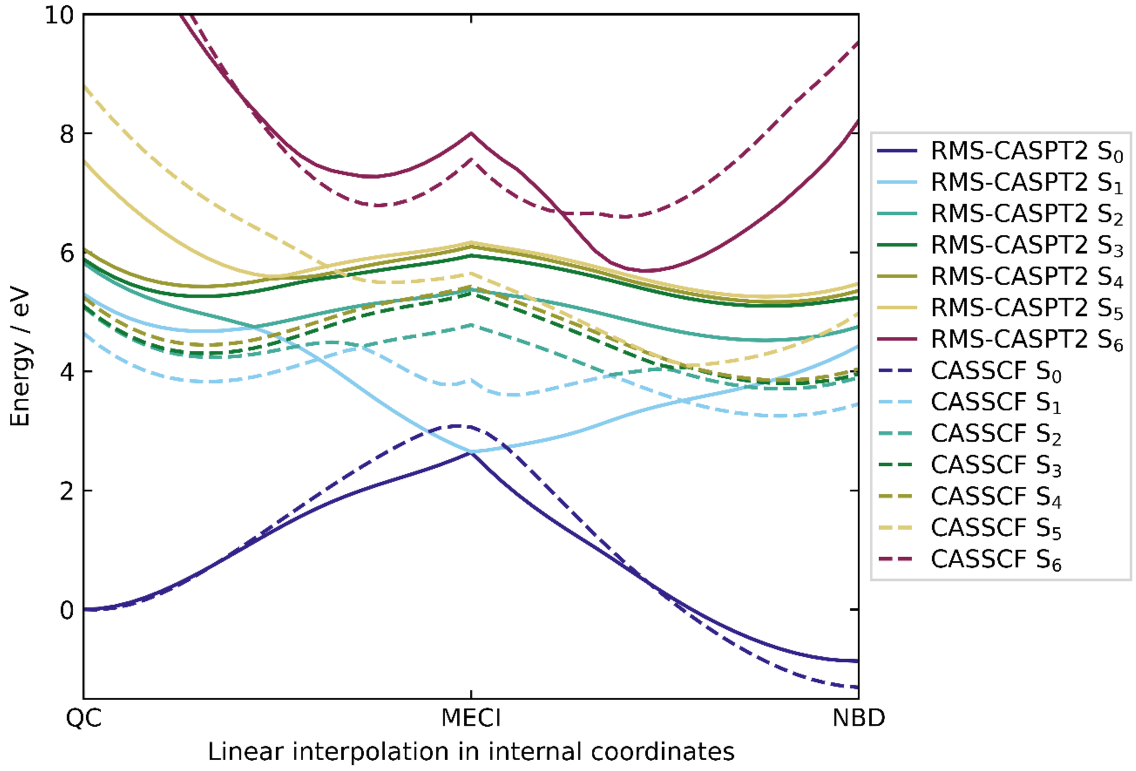

Supplementary Figure 14: Comparison of CASSCF and CASPT2. LIIC plot of RMS(9)-CASPT2(2,6) (solid lines) and SA(9)-CASSCF(2,6) (dashed lines) potential energy curves (PECs). The lowest seven states are shown, including the six states that participate in the dynamics and one additional state for comparison. The highest-energy state shown (S_6, maroon color) is of double excited character. It can be clearly seen that the Rydberg manifold, i.e., the comparatively 'flat' PECs between in the energy range $4 \leq E \leq 6$ eV, is systematically lower in energy when using CASSCF compared to RMS-CASPT2.

Previous studies (Valentini et al., 2020) have used a (4,8) active space to describe this system, containing four valence orbitals plus the 3s and three 3p Rydberg orbitals. To achieve better stability in the simulations and improve computational efficiency, we moved to a (2,6) active space, removing the highest and lowest energy valence orbitals. Pictures of these active space orbitals are shown in Supplementary Fig. 15.

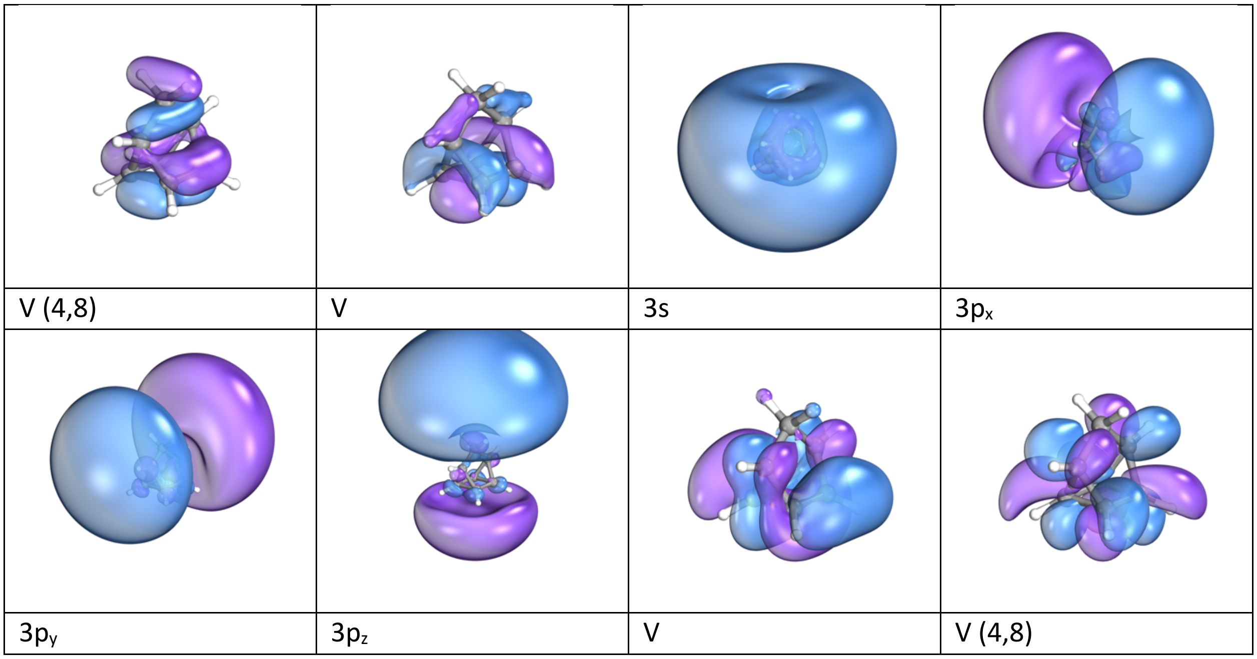

Supplementary Figure 15: **Active space orbitals calculated at QC.** The active space consists of 2 electrons in a space of 2 valence and 4 Rydberg orbitals. Extending the active space to include one additional pair of virtual and occupied orbitals creates the (4,8) active space of Valentini *et al*. (Valentini et al., 2020). The two orbitals that extend the (2,6) active space are identified by a (4,8) in their caption. The isosurface cutoff value has been adjusted such that 80% of the total density is shown for each orbital, and Rydberg orbitals are shown zoomed out to fully render their spatial extent. A rendering of the molecule is included in each frame. This figure is the same as Extended Data Fig. 5.

The RMS-CASPT2 PECs for both of these active spaces are shown in Supplementary Fig. 16. The agreement is good for most states, but the highest state, S_6, is described qualitatively differently by the (4,8) and (2,6) active spaces. This state is mostly of doubly-excited character and is only important for Rydberg dynamics on the NBD side of the dynamics, lying higher in energy in QC-like geometries. As the Rydberg dynamics is mostly constrained to the QC side, this state does not significantly affect the dynamics.

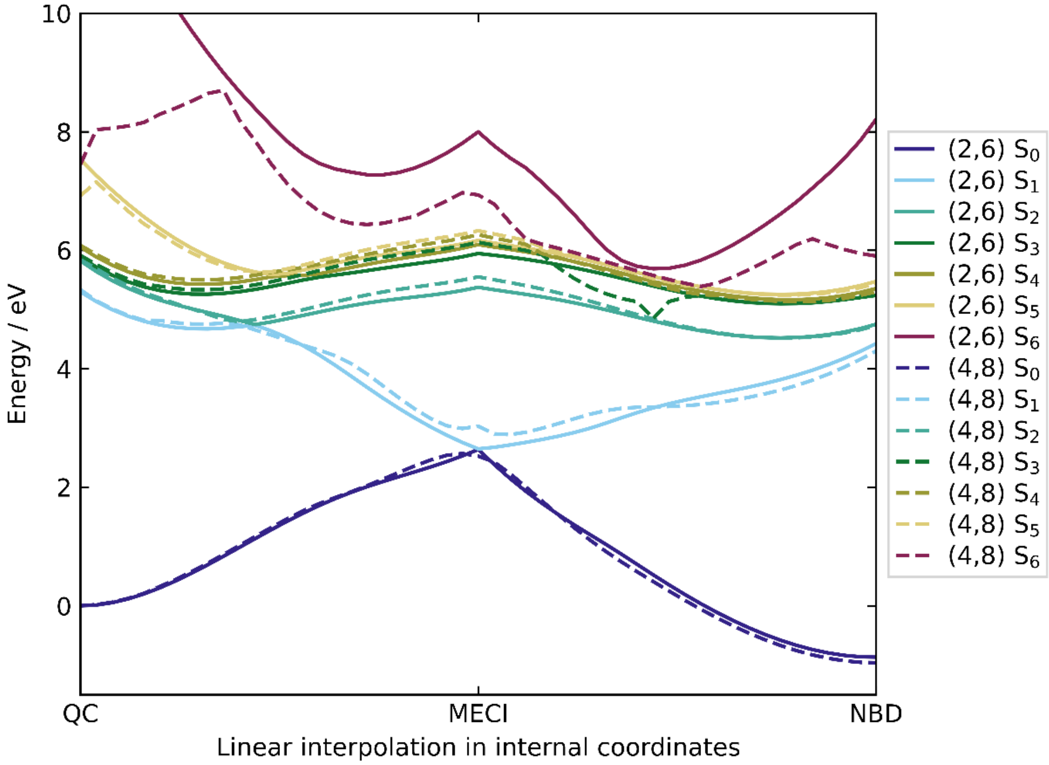

Supplementary Figure 16: Comparison of the (2,6) and (4,8) active space for CASPT2. Potential energy curves are shown along the LIIC for RMS-CASPT2 using either (2,6) (solid lines) or the (4,8) (dashed lines) active space, using the 6-31G*+D basis set. The first seven states are shown, which include the six dynamically active states and one further state for comparison. The (2,6) gives very similar PECs across the LIIC pathway for almost all the states. The only state incorrectly described in the (2,6) active space is the highest energy state with doubly excited character – shown as the solid maroon line. This is significantly lower in energy in the (4,8) active space (the dashed line running parallel), but lies significantly higher in energy at QC-like geometries. Its correct description is only important in the excited state manifold of NBD-like geometries, which are rarely visited in the current dynamics.

A proper description of Rydberg states requires a diffuse component to the basis set. The most common way to perform this is to use basis sets like aug-cc-pVDZ and 6-31++G, which add additional diffuse functions to every atom. This significantly increases the size of the basis, and hence the computational cost of the calculations. To address this, we used a technique like that described in Lorentzon *et al.* (Lorentzon et al., 1994). We optimize a contracted single S and P basis set function attached to the bridging carbon (in the CH_2 fragment) using primitives from Kaufmann *et al.* and added it as an additional segment to the standard 6-31G* basis set for that particular atom. The primitives and contractions of these functions are shown in Supplementary Table 5. We shall refer to this basis set as 6-31G*+D.

Supplementary Table 5: Basis sets primitives and contraction coefficients for the additional S and P basis functions (Lorentzon et al., 1994). This basis gives an acceptable level of agreement compared to standard basis sets for Rydberg calculations, e.g. aug-cc-pVDZ (see Supplementary Fig. 17). This provides a significant computational saving, allowing the calculations to use the dynamically correlated RMS-CASPT2 method.

S		P	
Prim.	Coeff.	Prim.	Coeff.
0.24624	0.61938966	0.042335	0.095483047
0.11253	-1.6065374	0.019254	-0.21148730
0.005858	0.62877974	0.009988	-0.87477491
0.003346	-1.2909713	0.005689	0.22484356
0.002948	1.5983647	0.003476	-0.47278922
0.001324	-0.12722174	0.002242	0.40679967
0.000893	0.6792952	0.001511	-0.23103729
0.000624	-0.16767218	0.001055	0.061411951

The comparison between 6-31G*+D and aug-cc-pVDZ is shown in Supplementary Fig. 17. There is an acceptable level of agreement between the two sets of PECs. The greatest difference is seen in terms of the relative description of the valence states and Rydberg manifold. This could contribute to the difference in Rydberg lifetimes observed in the paper and relates to the effective strength of Rydberg/valence coupling.

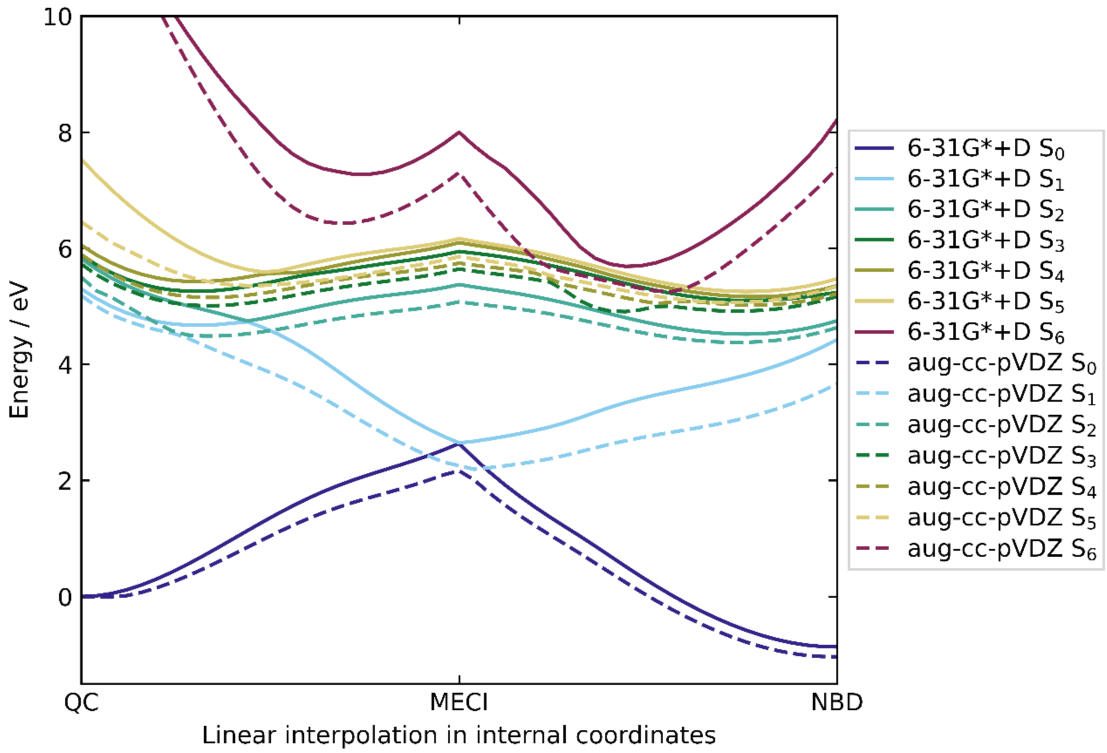

Supplementary Figure 17: Modified basis set. Comparison of the standard aug-cc-pVDZ basis set (dashed lines) and the modified 6-31G*+D basis set (solid lines) described in the caption to Supplementary Table 5. The comparison is made using the RMS(9)-CASPT2(2,6) method, limited to the six lowest-energy states, plus one more state for comparison. The description of the Rydberg states is quite consistent between the two methods, with some difference in the valence states noted. This difference could well contribute to the difference in theoretical and experimental Rydberg decay times.

2.3 Dynamics

As discussed in the Methods section of the main manuscript, the simulations employed FSSH as implemented in SHARC (Mai et al., 2018). On rare occasions, localized and minor convergence issues in the electronic structure calculations presented as localized dips in the potential energy surface. To mitigate these and thus avoid any potential stability issues in the dynamics simulations, we employed an adaptive step size. In this procedure, whenever the product of the root-mean-square of the gradient and the current time step became larger than a threshold value of 120 a.u, the time step was halved. The dynamics was propagated for 3 (short) time steps, and then returned to the starting value of 0.25 fs. This resolved the issue while having no effect on the other trajectories. For further analysis, all trajectories were mapped onto a regular grid with 0.5 fs time steps by stochastically selecting a single time step in each 0.5 fs time bin. This leads to limited 'roughness' of dynamical quantities when examined on a 0.5 fs scale but is undetectable on the longer time scales considered in this study.

2.4 Ionic state and photoelectron signal calculation

The ionic states were calculated using RMS(6)-CASPT2(1,6)/6-31G*+D – the equivalent method to the ground state with a single electron removed. Ionic states were calculated along each trajectory for a randomly selected time point in each 5 fs interval.

The photoelectron signal $S(E_{BE})$ is calculated by as:

$$S(E_{BE}) = \sum_{k}^{N_{traj}} \sum_{i}^{N_{ion}} \left|\langle i|\Psi_a^k\rangle\right|^2 g(E_{BE}, \Delta E_{ia}), \qquad (5)$$

where the first sum runs over all N_{traj} trajectories, and the second over the N_{ion} ionic electronic states. The $\left|\langle i|\Psi_a^k\rangle\right|^2$ is the squared Dyson-norm, which approximates the cross section, where $|i\rangle$ is the ion electronic wavefunction and $|\Psi_a^k\rangle$ the neutral active state a wavefunction for trajectory kN_{traj}. Finally, $g(E_{BE}, \Delta E_{ia})$ is a gaussian broadening function of fixed width, centred at the ΔE_{ia}, which is the energy difference between states i and a.

In the calculation, the orbitals of the ground state wavefunction were used as a set of starting guess orbitals. As the ionic calculation consists of a few purely valence states, while the neutral calculation consists of more states of both Rydberg and valence character, the state-averaging procedure tends to give relatively lower energies for the ions than the neutrals. This has the effect of giving lower than expected values for the ionization potentials. This can be seen when comparing the 3p TRPES signature, which is at 2.1 eV BE in the theory compared to 2.3 eV BE in the experiment. As these states are only involved in the calculation of photoionization cross sections, this discrepancy does not affect the observed dynamics.

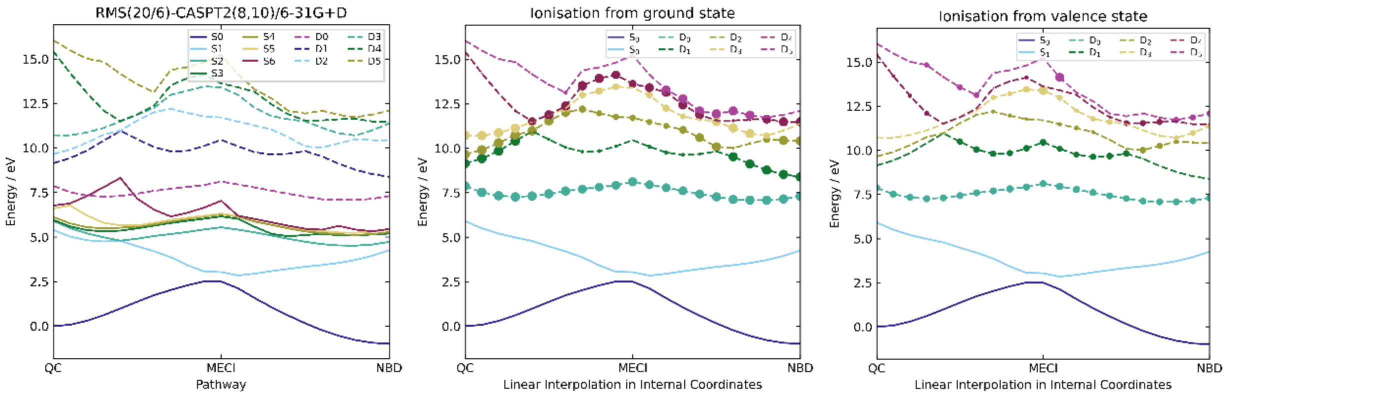

Supplementary Figure 18: Ionization cross-sections. LIICs with larger ionic state description. Left: Potential energy cut for larger active space with greater flexibility in the description of ionic states. Centre: Potential energy cuts, with photoionization cross-section from the ground state indicated by area of circle. Right: Potential energy cut, with photoionization cross-section from valence state indicated by area of circle.

In Supplementary Fig. 18, the earlier LIIC is plotted for a new method, RMS(20/6)-CASPT2(10,8)/6-31*G+D. This is an adaptation of our previous method that includes more occupied orbitals in the active space. It describes more ionic states than the current RMS-CASPT2(2,6) method but does not significantly

change the description of the states already included. The left panel in Supplementary Fig. 18 shows potential energy cuts for all states, while the center and right-hand panels only include the ground and valence excited state of the neutral, but also show the theoretical photoelectron cross-sections for transitions from these respective states, as a function of geometry along the LIIC.

The ground state (center panel) ionizes readily to multiple ionic states, but most peak at specific points along the pathway. Only ionization to the ground (D_0) ionic state is important around the S_1/S_0 MECI, but ionization to other D_n states contributes significantly around the respective ground state minima. While the theory used in the simulations reported in the main paper gives a good description of D_0 across all geometries, the D_1 (and higher) continua are poorly described at geometries close to the respective ground state minima. Notably, this mainly affects the signal from the ground state, which contributes less to the difference signal, while all ionization channels open for the excited state dynamics are well described in the simulations.

The valence state (right panel) ionizes to fewer states – the primary states being D_0 (across the whole pathway) and D_1 (around the conical intersection). These two states, which are well described in our current theoretical model (see Figure 3 in the main text), contain most of the signal from the TRPES simulations. This can be seen in Supplementary Fig. 19, which shows the theoretical TRPES signal for specific ionization continua: Ionization to D_0 (left panel, Supplementary Fig. 19) contributes the bulk of the signal and looks very similar to the ''hockey stick' picture presented and explained in the main text; it curves smoothly down from ~2 eV BE to the ground state at ~7 eV BE. The D_1 ionization (center panel, Supplementary Fig. 19) constitutes a smaller part of the signal and occupies a different part of the spectrum – high BE and early time. This appears upon motion of the wavepacket to the conical intersection, where the D_1 state is lower in energy.

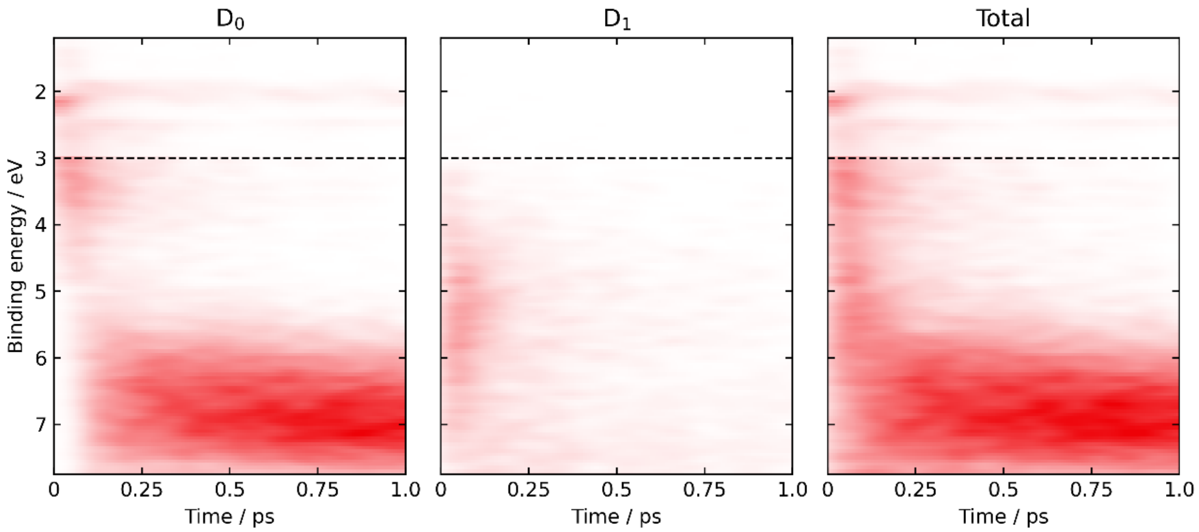

Supplementary Figure 19: Theoretical TRPES signal decomposition. Theoretical fast valence TRPES signal decomposed into ionization to D_0 (left panel), ionization to D_1 (center panel), and ionization to all modelled continua. All plots are shown on identical color bars and convolved with bivariate gaussians with 94 fs and 0.1 eV FWHMs.

Additionally, the Dyson norm cross-section is seen to be larger in the simulations for the Rydberg states relative to the valence states in comparison to the experiment. To explore the possibility of this being an

artefact of the Dyson norm calculation for the RMS-CASPT2(2/1,6) method, a larger RMS-CASPT2(4/3,8) Dyson norm calculation was performed on a representative sample of the trajectories, shown in Supplementary Fig. 20. The same active state index was used in both calculations. The RMS-CASPT2(4,8) calculation allows both more flexibility in describing the doublet wavefunctions and additional ionic states, and better describes the overlap between the ion and neutral wavefunctions. As can be seen in Supplementary Fig. 20, the RMS-CASPT2(4,8) calculation shows qualitatively brighter valence signal than the RMS-CASPT2(2,6). As such, the Rydberg region (BE < 3.0 eV) of the predicted TRPES spectra was re-scaled to 33% of its initial value to provide better qualitative agreement with the experimental spectrum.

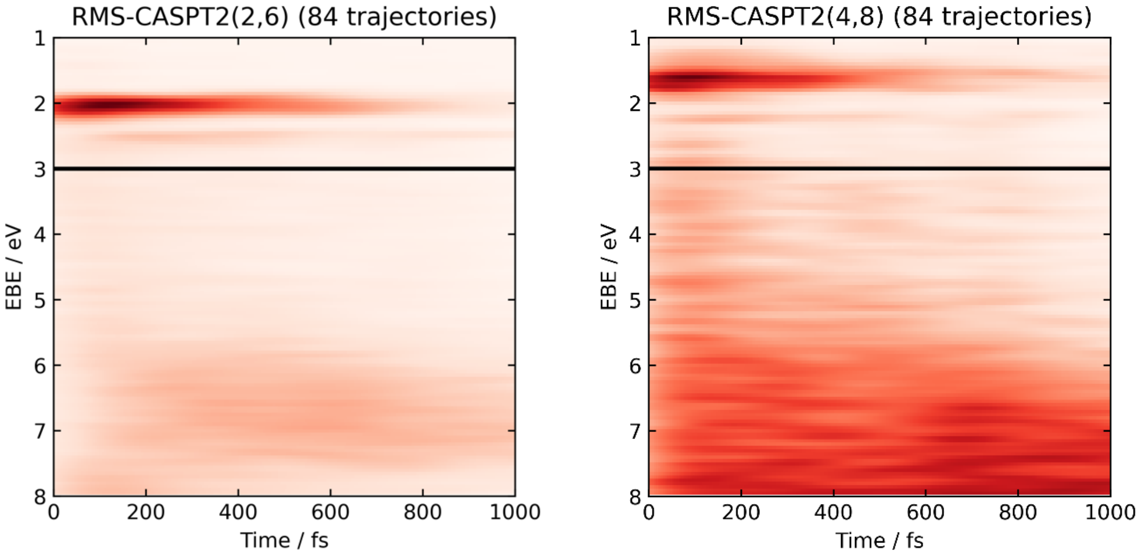

Supplementary Figure 20: **Time-resolved photoelectron spectra from simulations.** Comparison of RMS-CASPT2(2,6) (left) and RMS-CASPT2(4,8) (right) unscaled theoretical TRPES predictions for a representative sample of the dynamics. The Rydberg region is defined to have binding energy <3.0 eV, indicated with a black line. The RMS-CASPT2(4,8) clearly shows higher relative cross-sections than the RMS-CASPT2(2,6) calculations for the valence band in the range 3 < BE <8 eV but predicts the BEs of the 3p Rydberg bands less well (falling at ~1.7 eV BE), further from their experimental value of 2.3 eV BE.

2.5 Analysis of dynamics

Supplementary Fig. 21 shows the early-time simulated dynamics of the system, corresponding to Figure 4 (main text). The ultrafast decay of the valence pathway (top row of figures) can be seen to occur on a sub-100 fs timescale, with significant nuclear motion and population transfer being seen in panels A and B respectively. The Rydberg dynamics (bottom row), on the other hand, is much slower, with little vibrational motion and only minor population transfer.

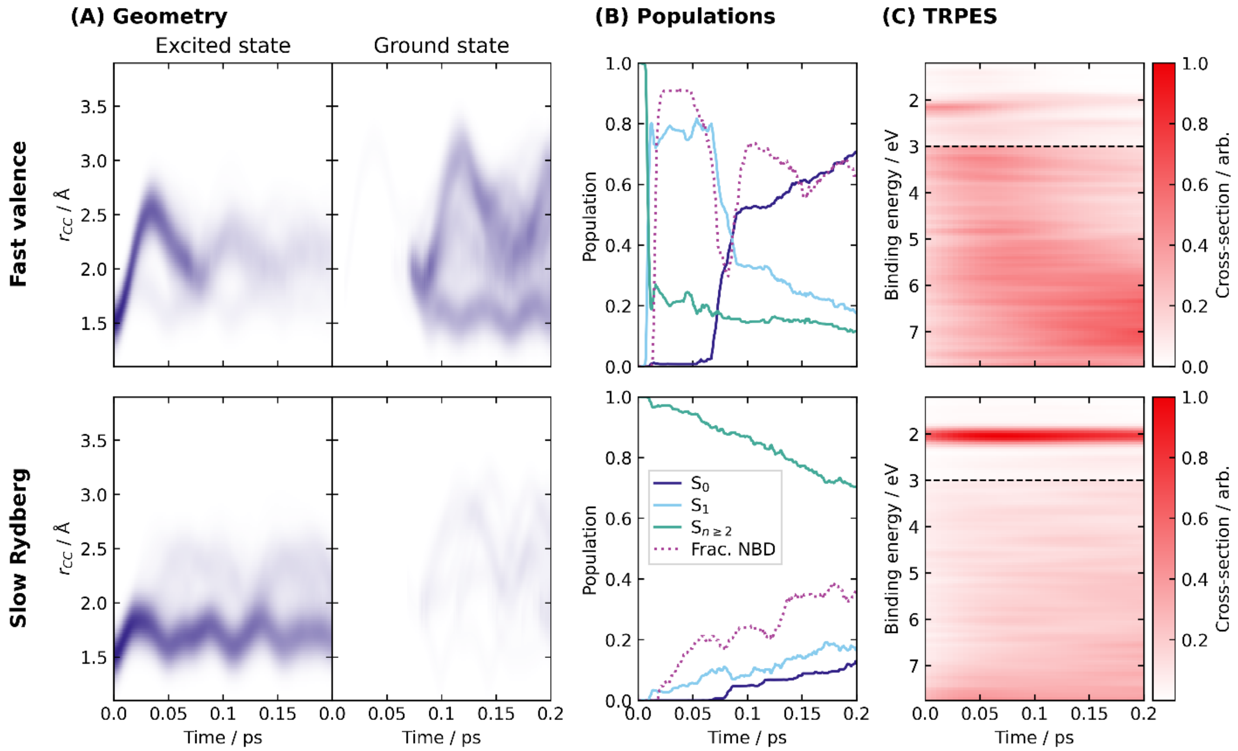

Supplementary Figure 21: **Early-time dynamics from simulations.** The same data as shown in Fig. 4 in the main text but zoomed in on the first 200 fs. This figure is the same as Extended Data Fig. 6.

Supplementary Fig. 22 shows the theoretical 'fast valence' TRPES signal in a series of integrated energy windows as a function of time. These cuts clearly illustrate the time-dependent BE profile – the peak of the photoelectron signal at higher BE shifts to longer time delays. This is even more apparent when considering ionization to just the D_0 continuum (left panel of Supplementary Fig. 19), which clearly shows the slanted BE vs time delay dependence.

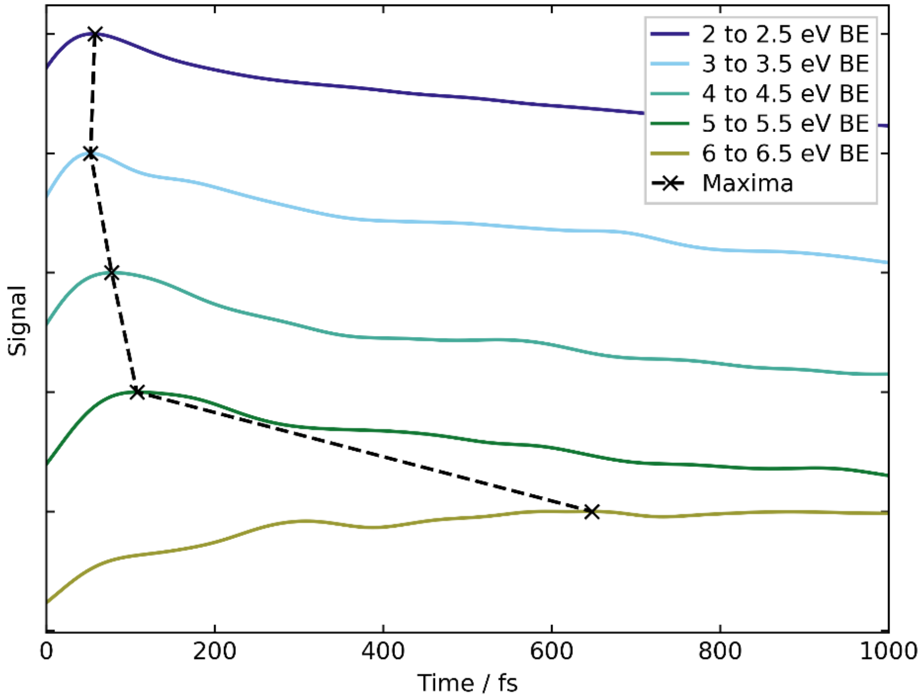

Supplementary Figure 22: Theoretical time dependence of TRPES signal. Theoretical TRPES lineouts, integrated over the binding energy window indicated. The maximum of each signal is marked with a cross and joined by the dashed line.

2.6 Absorption spectrum

The absorption spectrum of QC (Supplementary Fig. 23) was calculated using a nuclear ensemble with the Wigner sampling method. The S_1–S_0 band is largely associated with excitation to the 3s state, the S_2 adiabatic state corresponds almost entirely to the $3p_x$/V diabatic state and contributes to the *fast valence* pathway, whereas the S_3 and S_4 adiabatic states are well described in terms of the $3p_y$ and $3p_z$ diabatic states and contribute to the *slow Rydberg* pathway.

The experimental TRPES data showed dominant excitation to the 3p Rydberg states, with little direct excitation to the 3s state and no discernible excitation to 3d Rydberg states. Comparison with the calculated partial absorption cross-sections shown in Supplementary Fig. 23 then encouraged use of a delta pulse excitation at 5.75 ± 0.07 eV for the present dynamics simulations, which then involved 121 trajectories initiated on S_2 and 146 trajectories on the $S_{3/4}$ potentials.

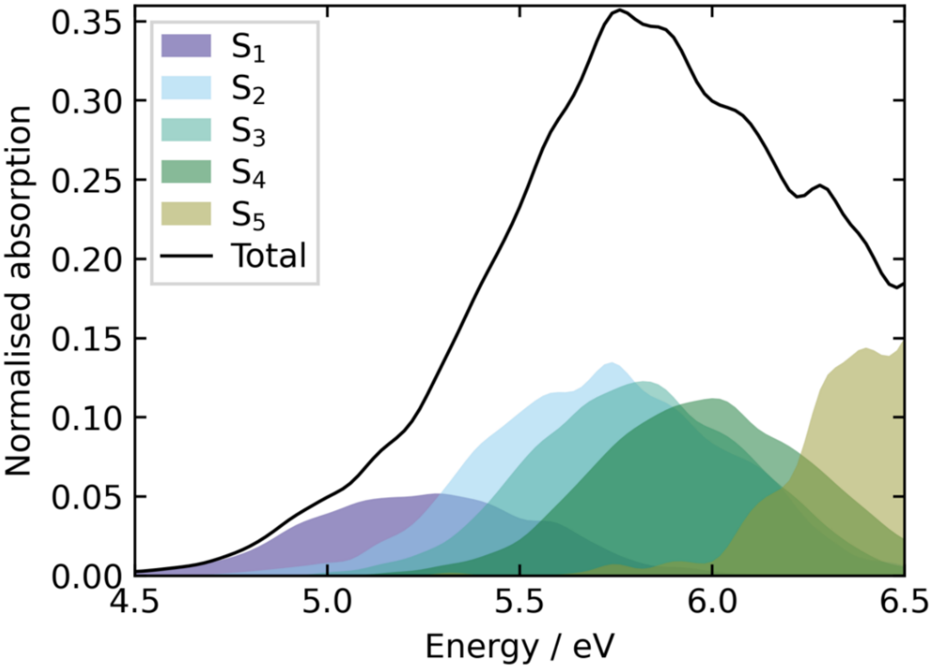

Supplementary Figure 23: **Absorption spectrum.** Wigner sampled absorption spectrum of QC using 10000 initial conditions broadened with a Gaussian (FWHM=0.1 eV). Calculated using geometries and energies at the RMS(9)-CASPT2(2,6)/6-31G*+D level. This figure is the same as Extended Data Fig. 7.

References

Allaria, E., Appio, R., Badano, L., Barletta, W. A., Bassanese, S., Biedron, S. G., Borga, A., Busetto, E., Castronovo, D., Cinquegrana, P., Cleva, S., Cocco, D., Cornacchia, M., Craievich, P., Cudin, I., D'Auria, G., Dal Forno, M., Danailov, M. B., De Monte, R., … Zangrando, M. (2012). Highly coherent and stable pulses from the FERMI seeded free-electron laser in the extreme ultraviolet. *Nature Photonics*, *6*(10), Article 10. https://doi.org/10.1038/nphoton.2012.233

Battaglia, S., & Lindh, R. (2021). On the role of symmetry in XDW-CASPT2. *The Journal of Chemical Physics*, *154*(3), 034102. https://doi.org/10.1063/5.0030944

Cobas, J. C., & Sardina, F. J. (2003). Nuclear magnetic resonance data processing. MestRe-C: A software package for desktop computers. *Concepts in Magnetic Resonance Part A*, *19A*(2), 80–96. https://doi.org/10.1002/cmr.a.10089

Fulmer, G. R., Miller, A. J. M., Sherden, N. H., Gottlieb, H. E., Nudelman, A., Stoltz, B. M., Bercaw, J. E., & Goldberg, K. I. (2010). NMR Chemical Shifts of Trace Impurities: Common Laboratory Solvents, Organics, and Gases in Deuterated Solvents Relevant to the Organometallic Chemist. *Organometallics*, *29*(9), 2176–2179. https://doi.org/10.1021/om100106e

Lorentzon, J., Malmqvist, PÅ., Fülscher, M. *et al.* A CASPT2 study of the valence and lowest Rydberg electronic states of benzene and phenol. *Theoret. Chim. Acta* **91**, 91–108 (1995). https://doi.org/10.1007/BF01113865

Mai, S., Marquetand, P., & González, L. (2018). Nonadiabatic dynamics: The SHARC approach. *WIREs Computational Molecular Science*, *8*(6). https://doi.org/10.1002/wcms.1370

Palmer, M. H., Coreno, M., de Simone, M., Grazioli, C., Aitken, R. A., Hoffmann, S. V., Jones, N. C., & Peureux, C. (2020). High-level studies of the ionic states of norbornadiene and quadricyclane, including analysis of new experimental photoelectron spectra by configuration interaction and coupled cluster calculations. *The Journal of Chemical Physics*, *153*(20), 204303. https://doi.org/10.1063/5.0031387

Pathak, S., Ibele, L. M., Boll, R., Callegari, C., Demidovich, A., Erk, B., Feifel, R., Forbes, R., Di Fraia, M., Giannessi, L., Hansen, C. S., Holland, D. M. P., Ingle, R. A., Mason, R., Plekan, O., Prince, K. C., Rouzée, A., Squibb, R. J., Tross, J., … Rolles, D. (2020). Tracking the ultraviolet-induced photochemistry of thiophenone during and after ultrafast ring opening. *Nature Chemistry*, *12*(9), Article 9. https://doi.org/10.1038/s41557-020-0507-3

Squibb, R. J., Sapunar, M., Ponzi, A., Richter, R., Kivimäki, A., Plekan, O., Finetti, P., Sisourat, N., Zhaunerchyk, V., Marchenko, T., Journel, L., Guillemin, R., Cucini, R., Coreno, M., Grazioli, C., Di Fraia, M., Callegari, C., Prince, K. C., Decleva, P., … Piancastelli, M. N. (2018). Acetylacetone photodynamics at a seeded free-electron laser. *Nature Communications*, *9*(1), Article 1. https://doi.org/10.1038/s41467-017-02478-0

Susnjar, P., Demidovich, A., Kurdi, G., Cinquegrana, P., Nikolov, I., Sigalotti, P., & Danailov, M. B. (2023). A novel common-path scheme for fourth harmonic generation by ultrashort laser pulses. *Optics Communications*, *528*, 129031. https://doi.org/10.1016/j.optcom.2022.129031

Svetina, C., Grazioli, C., Mahne, N., Raimondi, L., Fava, C., Zangrando, M., Gerusina, S., Alagia, M., Avaldi, L., Cautero, G., de Simone, M., Devetta, M., Di Fraia, M., Drabbels, M., Feyer, V., Finetti, P., Katzy, R., Kivimäki, A., Lyamayev, V., … Callegari, C. (2015). The Low Density Matter (LDM) beamline at FERMI: Optical layout and first commissioning. *Journal of Synchrotron Radiation*, *22*(3), 538–543. https://doi.org/10.1107/S1600577515005743

Travnikova, O., Piteša, T., Ponzi, A., Sapunar, M., Squibb, R. J., Richter, R., Finetti, P., Di Fraia, M., De Fanis, A., Mahne, N., Manfredda, M., Zhaunerchyk, V., Marchenko, T., Guillemin, R., Journel, L., Prince, K. C., Callegari, C., Simon, M., Feifel, R., … Piancastelli, M. N. (2022). Photochemical Ring-Opening Reaction of 1,3-Cyclohexadiene: Identifying the True Reactive State. *Journal of the American Chemical Society*, *144*(48), 21878–21886. https://doi.org/10.1021/jacs.2c06296

Valentini, A., van den Wildenberg, S., & Remacle, F. (2020). Selective bond formation triggered by short optical pulses: Quantum dynamics of a four-center ring closure. *Physical Chemistry Chemical Physics*, *22*(39), 22302–22313. https://doi.org/10.1039/D0CP03435E

Zangrando, M., Cocco, D., Fava, C., Gerusina, S., Gobessi, R., Mahne, N., Mazzucco, E., Raimondi, L., Rumiz, L., & Svetina, C. (2015). Recent results of PADReS, the Photon Analysis Delivery and REduction System, from the FERMI FEL commissioning and user operations. *Journal of Synchrotron Radiation*, *22*(3), 565–570. https://doi.org/10.1107/S1600577515004580

Zhou, X. J., Wannberg, B., Yang, W. L., Brouet, V., Sun, Z., Douglas, J. F., Dessau, D., Hussain, Z., & Shen, Z.-X. (2005). Space charge effect and mirror charge effect in photoemission spectroscopy. *Journal of Electron Spectroscopy and Related Phenomena*, *142*(1), 27–38. https://doi.org/10.1016/j.elspec.2004.08.004
